# Improved Properties of the Big Five Inventory and the Rosenberg Self-Esteem Scale in the Expanded Format Relative to the Likert Format

**DOI:** 10.3389/fpsyg.2019.01286

**Published:** 2019-06-04

**Authors:** Xijuan Zhang, Winnie Wing-Yee Tse, Victoria Savalei

**Affiliations:** Department of Psychology, University of British Columbia, Vancouver, CA, Canada

**Keywords:** big five inventory, Rosenberg Self-Esteem Scale, Expanded format, Likert format, order effect, method effect, acquiescence bias, negatively worded items

## Abstract

Previous research by Zhang and Savalei (2015) proposed an alternative scale format to the Likert scale format: the Expanded format. Scale items in the Expanded format present both positively worded and negatively worded sentences as response options for each scale item; therefore, they were less affected by the acquiescence bias and method effects that often occur in the Likert scale items. The major goal of the current study is to further demonstrate the superiority of the Expanded format to the Likert format across different psychological scales. Specifically, we aim to replicate the findings of Zhang and Savalei and to determine whether order effect exists in the Expanded format scales. Six psychological scales were examined in the study, including the five subscales of the big five inventory (BFI) and the Rosenberg self-esteem (RSE) scale. Four versions were created for each psychological scale. One version was the original scale in the Likert format. The other three versions were in different Expanded formats that varied in the order of the response options. For each scale, the participant was randomly assigned to complete one scale version. Across the different versions of each scale, we compared the factor structures and the distributions of the response options. Our results successfully replicated the findings of Zhang and Savalei, and also showed that order effect was generally absent in the Expanded format scales. Based on these promising findings, we encourage researchers to use the Expanded format for these and other scales in their substantive research.

## Introduction

Psychological scales are often written in the Likert format, in which respondents are asked how strongly they agree or disagree with the items. However, this scale format is known to have several problems (e.g., Ray, 1983; Saris et al., 2010; Sonderen et al., 2013; Wetzel and Greiff, 2018). First, the respondents may find the response options regarding the degree of agreement ambiguous (Saris et al., 2010). For example, consider a Likert item from the big five inventory (BFI): “I am someone who can be somewhat careless at times.” The response options are “disagree a little,” disagree a little,” “agree a little” and “agree strongly.” A respondent who picks “agree strongly” may mean (1) they are *very* careless at times, (2) they are somewhat careless *most of the time*, or (3) they are *definitely sure* that they can be careless at times. This ambiguity in the interpretation of the response options may increase measurement error, lowering the reliability and validity of the scale.

Another problem with the Likert format is that acquiescence bias, or the tendency for respondents to agree with an item indiscriminately regardless of their intended opinions (Ray, 1990), is commonly found in scales in the Likert format. To eliminate acquiescence bias, researchers constructed balanced scales by reversing the polarity of approximately half of the scale items, so that agreeing with these items indicates low endorsement of the construct being measured. Such reversed items are referred to as negatively worded (NW) items (DiStefano and Molt, 2006; Sonderen et al., 2013). The opposite of a NW item is a positively worded (PW) item; agreeing with a PW indicates high endorsement of the construct.

However, the advantages of using NW items have been questioned for at least two reasons. First, respondents who tend to acquiesce may agree with NW items as much as PW items, leaving contradictory and uninterpretable responses (Sonderen et al., 2013; Zhang et al., 2016). In theory, for a balanced scale, respondents’ total scores on the scale are not affected by these contradictory responses; however, the covariance structure of the scale will be affected (Savalei and Falk, 2014). Second, respondents can be confused by NW items or inattentive to the wording difference between intermittently ordered PW and NW statements, thereby missing the intended meaning of the items and giving erroneous responses (Sonderen et al., 2013; Zhang et al., 2016). These two issues may have caused the method effects seen in many balanced Likert scales (e.g., DiStefano and Molt, 2006, 2009; Lindwall et al., 2012; Tomas et al., 2013, 2015). Method effects are sources of variance that are unrelated to the construct being measured (DiStefano and Molt, 2006; Savalei and Falk, 2014; Vecchinone et al., 2014), thus posing a threat to the validity of measurement.

To solve the aforementioned problems with the Likert format, Zhang and Savalei (2015) proposed and investigated an alternative scale format called the Expanded format. Unlike the Likert format, in which respondents indicate how strongly they agree with a statement, an item in the Expanded format replaces each of the response options in the Likert scale (e.g., “disagree a little,” disagree a little,” “agree a little” and “agree strongly”) with a complete sentence, and asks respondents to pick one response option that best describes them. For example, the Likert item from the BFI (John et al., 2008), “I am someone who does a thorough job,” can be written in the Expanded format as follows:

– I am someone who does a very sloppy job.
– I am someone who does a somewhat sloppy job.
– I am someone who does a somewhat thorough job.
– I am someone who does a very thorough job.

The Expanded format can address the problems of the Likert format: first, the Expanded format reduces ambiguity of the response options by explicitly stating the meaning of each option; second, the Expanded format is not affected by acquiescence bias because it does not require respondents to agree or disagree with an item; third, method effects due to the polarity of Likert items are eliminated because items in the Expanded format are non-directional; and fourth, carelessness and confusion caused by the negation in some of the NW items are reduced because respondents need to pay attention to the subtle differences between the sentences to response to an Expanded item. As preliminary evidence of these benefits of the Expanded format, Zhang and Savalei (2015) found that psychological scales in the Expanded format had better (i.e., lower and more theoretically defensible) dimensionalities than scales in the Likert format. However, Zhang and Savalei (2015) only studied three scales. The main goal of the current study is to further investigate the benefits of the Expanded format by replicating Zhang and Savalei (2015)’s findings in scales both used and not used in their original study.

The second goal of our study is to examine whether order effect exists in Expanded format scales. Order effect is the tendency to choose a response option that is presented either first or last (e.g., Schwarz et al., 1992). Since the four response options in an Expanded item are listed in a column, order effect is one potential response bias. Several studies examined the existence of order effect in questionnaire items with multiple response options (e.g., McClendon, 1991; Schwarz et al., 1992). There are two major findings from this research. First, when an item has a list of response options, respondents are prone to choose the options at the top of the list; however, when respondents are given time to familiarize themselves with the response options, this is no longer the case (Krosnick and Alwin, 1987; Galesic et al., 2008). The order effect that is present when respondents do not have time to be familiar with the response options is likely caused by limited working memory or by the lack of cognitive effort (e.g., Krosnick and Alwin, 1987; Galesic et al., 2008). Because the Expanded format requires the respondents to do more reading, it is possible that order effect will be present in this format. The second major finding from this line of research is that when an item has a small number of response options (i.e., 2 to 4), respondents are more susceptible to order effect if they do not have predisposed opinions on the target issue (McClendon, 1991; Bergstrom et al., 2014). We suspect that this issue is not relevant for psychological scales that ask about the self.

The present study includes the two scales used in Zhang and Savalei (2015), which are the Conscientiousness subscale of the BFI (John et al., 2008) and the Rosenberg self-esteem (RSE) scale (Rosenberg, 1965), as well as four additional scales, which are the extraversion, agreeableness, neuroticism, and openness subscales of the BFI (John et al., 2008). The additional four scales, which are originally in the Likert format, were also converted to the Expanded format for the purposes of this study. The factor structures of each scale in the Likert format and in the Expanded format will be evaluated using confirmatory factor analyses (CFAs) and exploratory factor analyses (EFAs). We hypothesized that the data of the scale would follow a 1-factor structure better when the items are written in the Expanded format. We also hypothesized that a 2-factor model that separates the PW and NW items into two factors will result in an improvement in fit (over the fit of the 1-factor model) when the items are written in the Likert format, but that this will not be the case for the Expanded format. In other words, method effects that induce higher correlations among items of the same polarity and thus require a second factor to model, will not be present in the Expanded format. An exception, however, is the Openness scale which only has two NW items in the Likert format.

In order to investigate whether the Expanded format is susceptible to order effect, we also created several versions of each scale in the Expanded format, varying the order of the response options. If the Expanded scales are not affected by order effect, then the distributions of the response options for each item should be similar across the different versions, and the fit of CFA models should be similar across versions. We hypothesized that the scales in the Expanded format would generally not be affected by order effect because most people have strong predisposed beliefs about their own personality and self-esteem, and thus do not need to use cognitive shortcuts to respond to the BFI and the RSE scale.

## Materials and Methods

### Scale Construction

The original Likert version of the RSE scale and each of the BFI’s subscales (i.e., Conscientiousness, Extraversion, Neuroticism, Openness, and Agreeableness scales) were rewritten in the Expanded format. The RSE and Conscientiousness scales in the Expanded format were the same ones in Zhang and Savalei (2015). For each scale, four scale conditions were created (see Table 1 for an example):

(1) *Original version* – the original version of the scale in the Likert format;
(2) *Low-to-High version* – the Expanded format scale in which the item options are ordered from the option that indicates low endorsement of the construct to the option that indicates high endorsement of the construct;
(3) *High-to-Low version* – the Expanded format scale in which the item options are ordered from the option that indicates high endorsement of the construct to the option that indicates low endorsement of the construct; and
(4) *Half-Half version* – the Expanded format scale in which some items have options that are ordered from high endorsement of the construct to low endorsement of the construct, and some items have options that are ordered from low to high. For each scale, the PW items in the original Likert scale are the ones that are ordered from low to high; the NW items in the original Likert scale are the ones that are ordered from high to low.

**Table 1:** Two sample items for all versions.

	Sample PW Item	Sample NW Item
Original (Likert)	I am someone who is talkative.	I am someone who tends to be quiet.
Low-to-high (Expanded)	• I am someone who is very untalkative. • I am someone who is somewhat untalkative. • I am someone who is somewhat talkative. • I am someone who is very talkative.	• I am someone who tends to be very quiet. • I am someone who tends to be somewhat quiet. • I am someone who tends to be somewhat loud. • I am someone who tends to be very loud.
High-to-Low (Expanded)	• I am someone who is very talkative. • I am someone who is somewhat talkative. • I am someone who is somewhat untalkative. • I am someone who is very untalkative.	• I am someone who tends to be very loud. • I am someone who tends to be somewhat loud. • I am someone who tends to be somewhat quiet. • I am someone who tends to be very quiet.
Half-Half (Expanded)	• I am someone who is very untalkative. • I am someone who is somewhat untalkative. • I am someone who is somewhat talkative. • I am someone who is very talkative.	• I am someone who tends to be very loud. • I am someone who tends to be somewhat loud. • I am someone who tends to be somewhat quiet. • I am someone who tends to be very quiet.




*The response anchors for the original Likert version of the RSE Scale are “strongly disagree,” “somewhat disagree,” “somewhat agree,” and “strongly agree.” The response anchors for the Likert version of the BFI are “disagree strongly,” “disagree a little,” “agree a little,” and “agree strongly.” For the items in the Expanded versions, each participant was instructed to pick one of the four options that best describes him or her.*

### Participants and Procedure

Data were collected from undergraduate students who enrolled in psychology courses at the University of British Columbia (UBC). The dataset for this study can be found on Open Science Framework (OSF) platform webpage: https://osf.io/3r4mz/?view_only=115f60c0e2c143bb81ff906a12d4a7b7. Ethical approval was obtained through the UBC’s Behavioral Research Ethics Board. Participants completed an anonymous online survey that contained the BFI, the RSE scale, the life orientation test (LOT) (Scheier et al., 1994), the affect balance scale (ABS) (Bradburn, 1969) and a demographic questionnaire. The demographic questionnaire included regarding participants’ sex, age, ethnicity, birth country, and last term’s grade point average (GPA). For the RSE scale or each of the BFI’s subscales, participants were randomly assigned to complete one of the four versions. For the LOT, ABS and the demographic questionnaire, all participants completed the same version. A complete version of our survey can be found on our OSF platform webpage. A total of 1,184 participants participated in our study. The responses of 25 participants were deleted due to missing data, leaving 1,159 participants (see Supplementary Materials for the total number of respondents assigned to different versions of each scale). The average age was 20 years (*SD* = 2.55), and 78% were women. Participants were mainly East-Asian (51%) and Caucasian (24%).

### Descriptions of Scales

Complete versions for all scales are provided in the Supplementary Materials. For most items in the Expanded scale versions, the response options were created by adding a modifier to the original Likert items. For each scale version, we calculated the model-based reliability (e.g., Raykov, 1997) based on the 1-factor model. The model-based reliability makes less restrictive assumptions than the Cronbach’s alpha, which is generally an underestimate of the model-based reliability (for details, see Raykov, 1997).

We also conducted a preliminary assessment of the convergent validity of different versions of the scales by correlating them with criterion measures such as GPA, LOT, and ABS. The LOT measures generalized optimism (Scheier et al., 1994); the ABS measures positive affect (Bradburn, 1969). Past studies showed that conscientiousness is related to the GPA (e.g., Cheng and Ickes, 2009), and self-esteem and neuroticism are related to the LOT and ABS (e.g., Cheng and Furnham, 2003). Therefore, across different scale versions, we correlated the Conscientiousness scale with GPA, and the RSE and BFI’s Neuroticism scales with the ABS and LOT. The correlation coefficients are presented in Table 2. Overall, the correlations were general consistent across different versions of the same scale, indicating that changes in the scale format did not affect convergent validity. One exception is that the correlation between the Conscientiousness scale and GPA for the *High-to-Low* version (*r* = 0.34) is considerably higher than one for the *Original* version (*r* = 0.16).

**Table 2:** Correlations of the Conscientiousness, RSE and Neuroticism Scales with criterion measures.

	Conscientiousness and GPA	Neuroticism and LOT	RSE and LOT	Neuroticism and ABS	RSE and ABS
Original (Likert)	0.16	-0.66	0.74	-0.50	0.57
Low-to-High (Expanded)	0.29	-0.61	0.66	-0.41	0.60
High-to-Low (Expanded)	0.34	-0.64	0.70	-0.51	0.51
Half-Half (Expanded)	0.23	-0.67	0.65	-0.47	0.59


*All correlations are significant at *p* < 0.01.*

### Rosenberg Self-Esteem (RSE) Scale

The original RSE scale (Rosenberg, 1965) in the Likert format contains five PW items and five NW items, measured on a 4-point scale, where 4 corresponds to “strongly agree” and 1 corresponds to “strongly disagree” (see the Supplementary Materials for details of the scales). In the three Expanded versions (i.e., *Low-to-High, High-to-Low*, and *Half-Half*), each original Likert item is expanded into four response option sentences. The model-based reliabilities for the *Original, Low-to-High, High-to-Low*, and *Half-Half* versions are 0.94, 0.95, 0.94, and 0.94, respectively.

### Big Five Inventory (BFI)

The original BFI Conscientiousness scale contains five PW items and four NW items; the Extraversion and Neuroticism scales contain five PW items and three NW items; the Agreeableness scale contains five PW items and four NW items; the Openness scale contains eight PW items and two NW items (see the Supplementary Materials for details of the scales). The original items in the BFI are on a 5-point scale; however, we used a 4-point scale from “disagree strongly” to “agree strongly” in order to match the number of response options to that in the Expanded versions. For the Agreeableness scale, the model-based reliabilities for the *Original, Low-to-High, High-to-Low*, and *Half-Half* versions are 0.81, 0.84, 0.78, and 0.78, respectively; for the Conscientiousness scale, they are 0.85, 0.90, 0.88, and 0.83; for the Extraversion scale, they are 0.90, 0.92, 0.90, and 0.89; for the Neuroticism scale, they are 0.88, 0.88, 0.91, and 0.88; for the Openness scale, they are 0.85, 0.88, 0.88, and 0.85.

## Data Analysis

### Chi-Square Test of Homogeneity

For each scale item, we conducted a chi-square test of homogeneity to test whether the respondents’ endorsement distributions of the response options were the same across the *High-to-Low* and *Low-to-High* Expanded item versions (see Supplementary Materials for distributions of the response options). Because the *High-to-Low* and *Low-to-High* item versions only differ in the order of the response options, a significant chi-square test indicates the presence of order effect. We note that the chi-square test of homogeneity is a direct way of testing the presence of order effect at the *item level*. Factor analyses were done to examine the presence of order effect at the *scale level* (see below for details).

### Factor Analyses

For each version of each scale, we conducted both CFAs and EFAs to examine whether the scale in the Expanded format has better (i.e., lower and more theoretically defensible) dimensionalities relative to the scale in the Likert format and whether order effect exists in the scale with Expanded format at the scale level. In the case where the Expanded format scale does not have lower dimensionality, the EFAs will also help us explore alternative factor structures of the scale and find possible explanations for the unexpected result.

#### Confirmatory Factor Analyses (CFAs)

For CFAs, we fit two models to every version of each scale using the *lavaan* package (version 0.6-3) (Rosseel, 2012) in *R*. These two models are commonly used to study method effects in Likert scales (e.g., Marsh et al., 2010; Alessandri et al., 2011; Lindwall et al., 2012). The first model is a 1-factor model in which all items of a scale load on one factor. The second model is a 2-factor model in which all PW items in the original Likert scale load on one factor and all NW items load on the other factor, and the two factors are allowed to correlate (see sections “Rosenberg Self-Esteem Scale” and “Big Five Inventory” for the number of NW or PW items in each scale).

If the Expanded format can reduce the acquiescence bias and method effects often present in a Likert scale, then for the Likert scale version, the 1-factor model fit will be worse than the 2-factor model fit, whereas for the *Low-to-High* or *High-to-Low* Expanded version, the 1-factor model fit will be similar to the 2-factor model fit. If order effect is present in the Expanded scale versions, then in the *Half-Half* Expanded version, the order effect in the items that are ordered from low construct endorsement to high construct endorsement will be opposite of the order effect in the items that are ordered from high to low, thus causing the scale to not follow a one factor structure. In other words, the way order effect affects the Expanded scale’s factor structure is similar to how acquiescence bias affects the Likert scale’s factor structure. Therefore, if order effect is present, then the fit of the 1-factor model for the *Half-Half* version will be worse than the fit for the *Low-to-High* and *High-to-Low* Expanded versions.

The models were evaluated using five fit indices: (1) the test of exact model fit using the robust (mean-and-variance adjusted) chi-square statistic for ordinal data; (2) the comparative fit index (CFI), with the value of 0.90 or greater indicating a well-fitting model (Bentler, 1990); (3) the root mean square error of approximation (RMSEA), with the value of 0.08 or less indicating reasonable fit (Browne and Cudeck, 1993); (4) the standardized root mean square residual (SRMR), with the value of 0.08 or less indicating good fit (Hu and Bentler, 1999); (5) chi-square difference tests between the 1-factor and 2-factor models, using the method suggested by Satorra (2000) and implemented in *lavaan*.

#### Exploratory Factor Analyses (EFAs)

For EFAs, we conducted parallel analyses to all versions of all scales to determine the number of factors in each scale version using the *fa.parallel* function in the *psych* package (Revelle, 2015). Parallel analysis is an improved version of scree plot that can be used to determine the number of factors (Horn, 1965); its main advantage over the scree plot is that it incorporates sampling variability (Zwick and Velicer, 1986). Since scales in the Expanded format are not affected by acquiescence bias or method effects associated with the NW items, we hypothesized that the results of the parallel analyses should show that Expanded scales have fewer factors (ideally just one factor) than Likert scales.

If the Expanded scale does not follow the 1-factor structure well, it may indicate that the scale measures more than one substantive factors; in the case of the *Half-Half* Expanded version, it may also indicate that order effect is present. Therefore, in the case where an Expanded version of a particular scale had more than one factor based on the parallel analysis, we conducted further EFAs to investigate the scale’s factor structure by extracting the number of factors suggested by the parallel analyses using the *fa* function in the *psych* package. Based on these EFA results, a new CFA model was fit across all versions of the scale. We note that the results of these analyses are purely exploratory and should be confirmed in future studies. We perform these exploratory analyses to help find possible explanations why some scales in the Expanded format have poor fit under the 1-factor model or why they do not follow the 1-factor structure better than the scales in the Likert format. Apart from issues of method effects and acquiescence bias, some of the constructs may simply not be unidimensional.

## Results

Average item means and standard deviations for all versions of each scale are shown in Supplementary Materials. Generally, on average, item means were similar across the four versions of each scale. All versions of the scales in the Expanded format consistently had smaller standard deviations than the corresponding scales in the Likert version, except for the Agreeableness scale, which had similar standard deviations across the four versions. Moreover, the standard deviations of the three Expanded versions were very similar for all the scales. The reduction in the standard deviations of the Expanded items may be due to the reduction in the method effects of these items.

### Chi-Square Tests of Homogeneity

The *p*-value for each chi-square test of homogeneity is shown in Table 3. Most chi-square tests (i.e., 45 out of 54 chi-square tests) had non-significant *p*-values (*p* > 0.05). This result indicates that the distributions of the response options were similar across the *High-to-Low* and *Low-to-High* Expanded versions; therefore, most items were not affected by order effect. Inspecting the distributions of the response options of the nine items that have significant *p*-values, we found that respondents were more likely to pick an option when it was presented first, indicating the possible presence of an order effect in these items (see Supplementary Materials for all distributions of the response options). On the other hand, because many significance tests were conducted, some *p*-values may be significant by chance assuming that the distributions are the same across the *High-to-Low* and *Low-to-High* versions.

**Table 3:** *P*-values of chi-square test of homogeneity for all items in all scales.

	RSE	Conscientiousness	Extraversion	Neuroticism	Openness	Agreeableness
Item 1	0.06	0.31	0.48	0.09	0.09	0.87
Item 2	0.03^*^	0.00^*^	0.44	0.18	0.52	0.13
Item 3	0.02^*^	0.34	0.11	0.91	0.23	0.48
Item 4	0.61	0.43	0.48	0.30	0.31	0.41
Item 5	0.37	0.00^*^	0.97	0.39	0.64	0.01^*^
Item 6	0.11	0.27	0.88	0.60	0.56	0.40
Item 7	0.28	0.67	0.82	0.00^*^	0.39	0.01^*^
Item 8	0.00^*^	0.61	0.32	0.44	0.21	0.56
Item 9	0.68	0.93	N/A	N/A	0.66	0.54
Item 10	0.01^*^	N/A	N/A	N/A	0.53	N/A


*^∗^*p* < 0.05. For the RSE scale, items 3, 5, 8, 9, and 10 are NW in the Original version. For the Conscientiousness scale, items 2, 4, 5, and 9 in the Conscientiousness scale are NW in the Original version. For the Extraversion scale, items 2, 5, and 7 are NW in the Original version. For the Openness scale, items 5 and 7 are NW in the Original version. For the Agreeableness scale, items 1, 3, 5, and 8 are NW in the Original version.*

### Confirmatory Factor Analyses (CFAs)

The fit indices for the CFA models are presented in Table 4. The factor loadings and correlations for the CFA models are provided in the Supplementary Materials. In addition to the 1- and 2-factor models, based on a reviewer’s suggestion, we have also fit a factor model with all NW items loading on a method factor. Since the patterns of results for this method factor model are very similar to those for the 2-factor model, we will not discuss the results for this model in the following sections. For readers who are interested in the method factor model, we have provided the model’s fit summary in the Supplementary Materials.

**Table 4:** Summary of the fit indices for 1-factor and 2-factor models.

	1-factor model				2-factor model				
			
	χ^2^	CFI	RMSEA	SRMR	χ^2^	CFI	RMSEA	SRMR	Δχ^2^
**RSE**	(df = 35)				(df = 34)				(df = 1)
Original (Likert)	400.89	0.93	0.19	0.09	230.04	0.96	0.14	0.06	87.45 (*p* = 0.00)
Low-to-High (Expanded)	142.02	0.99	0.10	0.05	141.46	0.98	0.10	0.05	0.00 (*p* = 1.00)
High-to-Low (Expanded)	169.94	0.97	0.12	0.07	168.73	0.97	0.12	0.07	0.35 (*p* = 0.55)
Half-Half (Expanded)	184.15	0.97	0.12	0.06	166.43	0.97	0.12	0.06	16.89 (*p* = 0.00)
**Conscientiousness**	(df = 27)				(df = 26)				(df = 1)
Original (Likert)	135.63	0.91	0.12	0.08	97.33	0.94	0.10	0.07	24.36 (*p* = 0.00)
Low-to-High (Expanded)	108.99	0.96	0.10	0.06	107.56	0.96	0.10	0.06	1.93 (*p* = 0.16)
High-to-Low (Expanded)	80.95	0.96	0.08	0.06	78.57	0.96	0.08	0.06	2.74 (*p* = 0.10)
Half-Half (Expanded)	54.54	0.97	0.06	0.06	33.64	0.98	0.04	0.04	15.59 (*p* = 0.00)
**Extraversion**	(df = 20)				(df = 19)				(df = 1)
Original (Likert)	297.97	0.89	0.22	0.11	205.63	0.93	0.18	0.08	48.50 (*p* = 0.00)
Low-to-High (Expanded)	95.19	0.98	0.11	0.06	94.87	0.98	0.12	0.06	0.02 (*p* = 0.88)
High-to-Low (Expanded)	103.86	0.96	0.12	0.05	102.36	0.96	0.12	0.06	1.82 (*p* = 0.18)
Half-Half (Expanded)	111.64	0.95	0.13	0.06	118.86	0.95	0.14	0.06	3.34 (*p* = 0.07)
**Neuroticism**	(df = 20)				(df = 19)				(df = 1)
Original (Likert)	135.78	0.94	0.14	0.08	83.56	0.97	0.11	0.06	35.96 (*p* = 0.00)
Low-to-High (Expanded)	137.65	0.93	0.14	0.08	134.20	0.93	0.15	0.08	3.92 (*p* = 0.05)
High-to-Low (Expanded)	144.01	0.95	0.15	0.07	153.62	0.94	0.16	0.07	0.80 (*p* = 0.37)
Half-Half (Expanded)	145.13	0.92	0.15	0.08	140.53	0.92	0.15	0.08	5.44 (*p* = 0.02)
**Openness**	(df = 35)			
Original (Likert)	218.27	0.89	0.14	0.09	N/A	N/A	N/A	N/A	N/A
Low-to-High (Expanded)	339.00	0.85	0.17	0.13	N/A	N/A	N/A	N/A	N/A
High-to-Low (Expanded)	315.55	0.86	0.17	0.12	N/A	N/A	N/A	N/A	N/A
Half-Half (Expanded)	228.34	0.89	0.14	0.10	N/A	N/A	N/A	N/A	N/A
**Agreeableness**	(df = 27)				(df = 26)				(df = 1)
Original (Likert)	69.45	0.95	0.07	0.06	43.34	0.98	0.05	0.05	18.10 (*p* = 0.00)
Low-to-High (Expanded)	61.64	0.96	0.07	0.06	57.45	0.97	0.07	0.06	4.22 (*p* = 0.04)
High-to-Low (Expanded)	80.95	0.87	0.08	0.08	53.24	0.93	0.06	0.06	16.46 (*p* = 0.00)
Half-Half (Expanded)	145.76	0.85	0.13	0.10	95.32	0.91	0.10	0.08	31.86 (*p* = 0.00)


*All χ^*2*^ tests are significant at *p* < 0.01. All CFA models are estimated using *lavaan* (version 0.6-3); the diagonally weighted least squares estimator with robust corrections was used (i.e., estimator = “WLSMV” in *lavaan*). because all items were measured on a 4-point scale and thus are treated as ordinal data (Rhemtulla et al., 2012). Since the Openness scale had only two NW items (i.e., two items cannot form a factor), the 2-factor model cannot be fit to the scale. Δ*χ^*2*^* was calculated according to Satorra’s (2000) suggestion for calculating chi-square difference test using the Satorra-Bentler scaled chi-square.*

#### One-Factor Model

We first examine the fit of the 1-factor model. Overall, the 1-factor model fit most versions of the RSE, Conscientiousness, Neuroticism and Extraversion scales by CFI and SRMR, but the RMSEA results were more mixed. For the Agreeableness scale, all three fit indices indicated good fit for the Likert version of the scale and the *Low-to-High* Expanded version but not for the *High-to-Low* and *Half-Half* Expanded versions. For the Openness scale, the 1-factor model did not fit the data from any version of this scale, according to all three fit indices.

Regarding the main research goal of replicating the results of Zhang and Savalei (2015), for the Likert version of the RSE, Conscientiousness and Agreeableness scales, the 1-factor model fit was worse than that for the Expanded versions. For the Neuroticism and Openness scales, the 1-factor model fit was very similar across all versions. For the Agreeableness scale, the 1-factor model fit for the Expanded versions was worse than the fit for the Likert version, thus showing a result pattern opposite to our hypothesis.

Regarding the second research goal of examining order effect in the Expanded scales, when it comes to the different versions of the Expanded format, for the RSE, Conscientiousness, Extraversion and Neuroticism scales, the fit was similar across the three versions, indicating the absence of order effect. However, for the Agreeableness scale, the fit for the *Half-Half* version was worse than for other versions, implying the possible presence of order effect. In short, the Expanded versions of most of the scales (i.e., the RSE, Conscientiousness and Extraversion scales) had better 1-factor model fit than the Likert versions, and order effect was absent in all scales except the Agreeableness scale.

#### Two-Factor Model

The 2-factor model posits two correlated factors (one among PW items and one among NW items). Since the Openness scale had only two NW items (i.e., two items cannot form a factor), we did not fit the 2-factor model to the scale. The 2-factor model for all versions of all five scales achieved good fit by CFI and SRMR but not by RMSEA.

Regarding the main research goal, as predicted, for the Likert versions of all scales, the 2-factor model fit was significantly better than the 1-factor model fit (i.e., very large and significant chi-square difference between the 1-factor and 2-factor models; higher CFI, and lower RMSEA and SRMR); in contrast, for the Expanded versions, the 2-factor model fit was generally similar to the 1-factor model fit (i.e., mostly smaller and insignificant chi-square differences between the 1-factor and 2-factor models; similar CFI, RMSEA and SRMR values). In addition, relative to the *Original* versions, the Expanded versions generally had much higher correlations between the two factors, further supporting the hypothesis that the Expanded versions are better modeled by the 1-factor model (see the Supplementary Materials for factor correlations for all scale versions). The major exception is the Agreeableness scale. For both *High-to-Low* and *Half-Half* Expanded versions of the Agreeableness scale, the 2-factor model fit was much better than the 1-factor model fit, and the factor correlations were lower than the one for the *Original* version.

Regarding the second research goal, for the RSE, Conscientiousness, Extraversion and Neuroticism scales, the fit indices for the 2-factor model among the three Expanded versions were similar; however, for the Agreeableness scale, the fit for the *High-to-Low* and *Half-Half* versions was worse than the fit for the *Low-to-High* version, indicating possible order effect in the Agreeableness scale. Therefore, except for the Agreeableness scale, the results were consistent with our hypotheses of the two research goals.

### Exploratory Factor Analyses (EFAs)

The results of parallel analyses for each scale are provided in Supplementary Materials. For the RSE, Conscientiousness and Extraversion scales, one factor was suggested for the Expanded versions; two factors were suggested for the Likert format. These results are consistent with our hypothesis that the scale in the Expanded format has better factor structure with lower dimensionality relative to the scale in the Likert format.

However, for the Neuroticism scale, two factors were suggested for the *Low-to-High* and *Half-Half* Expanded versions but one factor was suggested in the other two versions; for the Openness scale, three factors were suggested for all four versions; and for the Agreeableness scale, three factors were suggested for the *High-to-Low* and *Half-Half* Expanded versions; two factors were suggested for the other two versions. Since the parallel analyses suggested that the Expanded versions of the Neuroticism, Openness and Agreeableness scales have more than one factor (i.e., contrary to our hypothesis), we conducted further EFAs to explore the structure of these scales (see Supplementary Materials for detailed results). Based on the parallel analyses results, we extracted two factors for the Neuroticism scale and three factors for the Agreeableness and Openness scales.

For the Neuroticism scale, the loading patterns for the Likert version were more based on item polarity whereas the patterns for the Expanded versions were more based on item content. For the Likert version, Factor 1 was mostly defined by the PW items whereas Factor 2 was mostly defined by the NW items. For the three Expanded versions, Factor 1 was defined by Items 2, 3, 4, 7, and 8; Factor 2 was defined by Items 1, 5 and 6 (see Supplementary Materials for the item content). Inspecting the scale’s item content, we found that the items loading on Factor 1 of the Expanded versions are more related to anxiety whereas the items loading on Factor 2 are more related to depression. Therefore, a suitable alternative model based on the item content is a 2-factor model with Items 2, 3, 4, 7, and 8 loading on Factor 1 (i.e., the anxiety factor), and Items 1, 5 and 6 loading on Factor 2 (i.e., the depression factor).

For the Openness scale, the loading patterns across the four versions were quite consistent. Factor 1 was defined by Items 1, 2, 3 and 5, which are related to creativity; Factor 2 was defined by Items 4, 8, and 9, which are related to artistic talent; Factor 3 was defined by Items 6, 7, and 10, which are about the tendency to be a deep thinker. Therefore, based on the item content, a suitable alternative model for the Openness scale is a 3-factor model. Items 1, 2, 3, and 5 form Factor 1, representing creativity; Items 4, 8, and 9 form Factor 2, representing artistic talent; Items 6, 7 and 10 form Factor 3, representing the tendency to be a deep thinker.

For the Agreeableness scale, when three factors were extracted, there were not a lot of consistent and interpretable patterns across the versions; the only consistent pattern is that Items 3 and 8 always loaded on Factor 2. Inspecting the item content, we found that Items 3 and 8 are both about being respectful to others. Therefore, a suitable alternative model based on the item content of the Agreeableness scale is a 1-factor model with a correlated residual between Items 3 and 8.

#### Alternative CFA Models

Based on the further EFAs, we came up with an alternative confirmatory factor model for each of the Neuroticism, Openness and Agreeableness scales. The model fit indices for these alternative models are presented in Table 5. The factor loadings for each model are provided in Supplementary Materials.

**Table 5:** Summary of the fit indices for the alternative models for the extraversion, neuroticism, openness, and agreeableness scale.

	χ^2^	CFI	RMSEA	SRMR
**Neuroticism**	(df = 19)			
Original (Likert)	132.85	0.94	0.14	0.07
Low-to-High (Expanded)	73.94	0.97	0.10	0.06
High-to-Low (Expanded)	97.62	0.97	0.12	0.06
Half-Half (Expanded)	89.47	0.96	0.11	0.06
**Openness**	(df = 32)			
Original (Likert)	64.88	0.98	0.06	0.05
Low-to-High (Expanded)	55.82	0.99	0.05	0.05
High-to-Low (Expanded)	60.31	0.99	0.06	0.05
Half-Half (Expanded)	61.76	0.98	0.06	0.05
**Agreeableness**	(df = 26)			
Original (Likert)	57.26	0.96	0.07	0.06
Low-to-High (Expanded)	44.25	0.98	0.05	0.05
High-to-Low (Expanded)	65.61	0.90	0.07	0.07
Half-Half (Expanded)	86.26	0.93	0.09	0.07


*All χ^*2*^ tests are significant at *p* < 0.01. All CFA models are estimated using *lavaan* (version 0.6-3); the diagonally weighted least squares estimator with robust corrections was used (i.e., estimator = “WLSMV” in *lavaan*) because all items were measured on a 4-point scale and thus are treated as ordinal data (Rhemtulla et al., 2012).*

For all versions of the Openness and Agreeableness scale, the alternative models had achieved good fit according to all three fit indices but for all versions of the Neuroticism scale, the alternative model had good fit according to the CFI and SRMR but not RMSEA. Comparing to the original 1-factor model, the alternative model for each scale had equal or better fit for all versions. A noticeable result is that for all versions of the Openness scale, the alternative model had much better fit than the original 1-factor model. For the Likert versions of the Neuroticism and Agreeableness scales, the fit of the alternative models was slightly worse than that of the original 2-factor model; however, for the Expanded versions of these scales, the fit of the alternative model was better than that of the original 2-factor model. Overall, the alternative models had good fit across all versions of all scales.

## Discussion

The current study had two goals:(1) to replicate Zhang and Savalei (2015) results on the superiority of the Expanded format relative to the Likert format on different psychological scales; and (2) to examine whether order effect exists in the Expanded format scales. We hypothesized that relative to scales in the Likert format, scales in the Expanded format would not be as susceptible to acquiescence bias and method effects, and thus have a more parsimonious factor structure (i.e. better fit of the 1-factor model). We also hypothesized that scales in the Expanded format would not be affected by order effect.

Our CFA results were mostly consistent with our hypothesis for the main research question. For the RSE, Conscientiousness and Extraversion scales, we were successful at replicating the findings of Zhang and Savalei (2015). For each of these scales, parallel analyses showed the Expanded versions followed one factor structure, but the Likert version followed two factor structure. CFAs also showed that the 1-factor model fit for the Expanded versions was better than that for the Likert version. Furthermore, for the Likert version of these scales, the 2-factor model fit was consistently and significantly better than the 1-factor model fit; in contrast, for the Expanded versions, the 2-factor model fit was similar to the 1-factor model fit. Therefore, we conclude that a scale in the Expanded format generally has a more parsimonious factor structure than the same scale in the Likert format.

Our hypothesis for the second research question was confirmed by the chi-square tests of homogeneity and by most CFA results. Out of the 54 chi-square tests of homogeneity testing whether item distributions were consistent across the three Expanded versions, forty-five tests were not significant, indicating the absence of order effect at the item level. For five of the six scales (i.e., all scales except the Agreeableness scale), the 1-factor model fit was relatively similar across the Expanded versions which varied only in the order of the response options, indicating the absence of order effect at the scale level. These results together suggest that scales in the Expanded format are generally not affected by order effect.

For the Openness scale, although the 1-factor model fit for the Expanded versions was not better than the fit for the Likert version, this result is not unexpected because the Likert version only has two NW items out of the ten items (see section “Introduction”). The 1-factor model fit for all versions of the Openness scale was poor. This result is consistent with the past research showing that the Openness scale is the most unreliable and problematic scale in the BFI (e.g., Gosling et al., 2003; Rammstedt and John, 2006). For example, Gosling et al. (2003) found that the Openness scale fared least well in the evaluation of convergent and discriminant validity, test-retest reliability, and convergence between self- and observer-ratings. Moreover, Soto and John (2017) claimed that the openness construct is composed of three smaller trait components, which are creative imagination, aesthetic sensitivity and intellectual curiosity. These three trait components roughly match onto the creativity factor, the artistic talent factor, and the tendency-to-be-a-deep-thinker factor found in our Openness scale. Consistent with this past research, when we fit an alternative model separating these three openness component traits, the model fit for the scale in the Likert and Expanded formats was excellent.

However, there were several result patterns that were inconsistent with our hypotheses. First, for nine items across the six scales, the chi-square tests of homogeneity were significant, indicating the possible presence of order effects in these items. For these items, respondents were more likely to pick an option when it was presented at the beginning of the list of response options. Perhaps, these items are more ambiguous than other items, making respondents more likely to use cognitive heuristics to answer them. Second, for the Neuroticism scale, the 1-factor model fit for the Expanded versions was not better than that for the Likert version. This result may be caused by the fact that the Neuroticism scale does not follow a 1-factor structure based on the item content. More specifically, from the EFAs and the inspection of the item content, we can see that Items 1, 5, and 6 are more related to depression whereas the other items are more related to anxiety. Past research has shown that depression and anxiety are two overlapping but distinctive constructs (e.g., Soto and John, 2017). In the Likert version, the three depression items do not have the same polarity: Items 1 and 6 are PW, and Item 5 is NW. As a result, for the Likert version, the correlations among the depression items may be similar to the correlations among the three NW items (Items 2, 5, and 7) because the depression items shared similar content but not the same polarity whereas the NW shared the same polarity but not similar content. This interaction between the item content and item polarity may have made the Likert version of the scale follow a one-factor structure better than it should. On the other hand, for the Expanded versions, the items do not have polarities; therefore, due to similar content, the correlations among the depression items may be higher than those between the depression and anxiety items. This may explain why the Likert version of the Neuroticism scale follows a one-factor structure better than the other Expanded versions. Indeed, when we fit an alternative model that separated the depression items from the anxiety items, the model fit for the Expanded versions was better than the fit for the Likert version.

The other result patterns inconsistent with our hypotheses are related to the Agreeableness scale. For this scale, the 1-factor model fit for the *Half-Half* and *High-to-Low* Expanded versions was much worse than the fit for the Likert version and the *Low-to-High* Expanded version. Also, for the *Half-Half* and *High-to-Low* versions, the 2-factor model fit was much better than the 1-factor model fit. We offer few possible explanations for these results. First, order effect may exist in the Expanded versions of the scale. The fact that the *Half-Half* Expanded version had the worst 1-factor model fit among the three Expanded versions supports this claim. If order effect exists in the Agreeableness scale, then in the *Half-Half* version, the order effect on the items that are ordered from low construct endorsement to high construct endorsement will be opposite of the order effect on the items that are ordered from high to low, thus causing the scale to not follow a one-factor structure. However, this explanation does not address why the 1-factor model fit for the *High-to-Low* Expanded version was much worse than that for the *Low-to-High* Expanded version. In addition, when we added a correlated residual between two items with similar content to the 1-factor model (i.e., fitting the alternative model for the Agreeableness scale), the model fit for the *Half-Half* version improved greatly. This result shows that the misfit of the *Half-Half* version is mainly due to a misspecification based on the item content, not due to order effect. Therefore, order effect does not fully explain the inconsistent result patterns.

The second explanation for these results is that the low validity and reliability of the Agreeableness scale made it hard to replicate the model fit results across the different conditions. Many studies (e.g., Gosling et al., 2003; Rammstedt and John, 2006) on the BFI show that the Agreeableness scale is the second most unreliable and problematic scale in the BFI (i.e., the first one is the Openness scale). Rammstedt and John (2006) consistently found that the Agreeableness scale had low test-retest reliability, and low convergent validation with the scales in the Revised NEO Personality Inventory. Consistent with these past research findings, on average, the Agreeableness scale had relatively low reliabilities compared to the other scales in our study (see section “Materials and Methods”). Together, these results suggest that agreeableness is not a well-defined construct; therefore, the Agreeableness scale may contain other substantive or method factors that are independent of the scale format and are attributable to our inconsistent findings for this scale.

### Limitation and Future Research

Our study has several limitations. First, we fit three alternative models to the Neuroticism, Openness and Agreeableness scales because the Expanded versions of these scales do not appear to follow a one-factor structure. These three models are derived from our EFAs; thus, it is unclear whether the results of these three models can be generalized to other datasets. Future research should replicate these results, prioritizing our final exploratory models as confirmatory *a priori*. Second, future research should investigate some of the possible reasons for the unexpected results from the Agreeableness and Neuroticism scales. A follow-up study could explore whether the factor structure of the Agreeableness scale in the *Half-Half* or *High-to-Low* Expanded format will improve if the scale is made more reliable.

Third, in this study, we only examined the scales’ convergent validity across different scale formats in limited ways. One challenge of investigating whether the format of a scale affects its validity is that the format of the criterion measure may interact with the scale format being studied. For example, suppose we want to study how the scale format (Likert vs. Expanded) affects the convergent validity of the RSE scale. If the criterion measure is also a self-report scale in the Likert format, then the Likert RSE scale may show similar or higher correlation with the criterion measure relative to the Expanded RSE scale, because the Likert RSE scale shares the same format as the criterion measure. Perhaps, the respondent exhibits the same amount of acquiescence bias to both the Likert RSE scale and the criterion measure, inflating the raw correlation between them. One way to combat this challenge is to find criterion measures that are not self-report. Therefore, in future studies, we plan to examine the validities of different versions of the BFI and the RSE scale through self-other agreement method.

Finally, over the past decades, other alternative scale formats have been proposed to address the problems with the Likert format (e.g., Wong et al., 2003; Saris et al., 2010). For example, the Item-Specific format proposed by Saris et al. (2010) replaces the Likert format’s generic response options regarding the degree of agreement with options tailored specific to each item (see Saris et al., 2010 for example items). Wong et al. (2003) also proposed an alternative format with options tailored specific to each item, but in their format, only the two extreme options were labeled in words: the middle options were labeled in numbers (see Wong et al., 2003 for example items). Items in both of these alternative formats will be shorter than those in the Expanded format; therefore, these two formats may be better for the scales with more items. In the future study, we plan to compare the Expanded format to other alternative scale formats.

In conclusion, our study confirmed that the Expanded scales generally have the same or better (i.e., smaller) dimensionalities than the Likert scales. The one potential problem of the Expanded format, order effect, was generally found absent in the Expanded scales. Based on these promising findings, we encourage other researchers to change existing Likert scales into the Expanded format and use Expanded format scales in their substantive research.

## Data Availability

All datasets generated for this study are included in the manuscript and/or the Supplementary Files.

## Ethics Statement

This study was carried out in accordance with the recommendations of the guidelines outlined by the University of British Columbia (UBC)’s Behavioral Research Ethics Board with written informed consent from all subjects. All subjects gave written informed consent in accordance with the Declaration of Helsinki. The protocol was approved by the UBC’s Behavioral Research Ethics Board.

## Author Contributions

XZ designed the study. XZ and WW-YT analyzed the data for the study. All authors were involved in the writing of the manuscript.

## Conflict of Interest Statement

The authors declare that the research was conducted in the absence of any commercial or financial relationships that could be construed as a potential conflict of interest.
