# Supplementary material for: Improved Properties of the Big Five Inventory and the Rosenberg Self-Esteem Scale in the Expanded Format Relative to the Likert Format
Source: Front Psychol. 2019 Jun 4;10:1286. doi: 10.3389/fpsyg.2019.01286 (PMC6558198; doi:10.3389/fpsyg.2019.01286)
Supplement: Supplementary file 1 [file Table_1.DOCX]

**Table 1: Total Number of the Respondents in Each Version of All Scales.**

|  | **Original/Likert** | **Low-to-High** | **High-to-Low** | **Half-Half** |
| --- | --- | --- | --- | --- |
| **Rosenberg Self-Esteem** | 289 | 292 | 289 | 289 |
| **Conscientiousness** | 290 | 290 | 290 | 289 |
| **Extraversion** | 293 | 291 | 289 | 286 |
| **Neuroticism** | 292 | 291 | 288 | 288 |
| **Openness** | 291 | 290 | 289 | 289 |
| **Agreeableness** | 286 | 293 | 299 | 281 |

**Table 2. Descriptive statistics for the scales.**

| **Average Item Mean** | | | | |
| --- | --- | --- | --- | --- |
|  | **Original** | **Low-to-High** | **High-to-Low** | **Half-Half** |
|  | **(Likert)** | **(Expanded)** | **(Expanded)** | **(Expanded)** |
| **RSE** | 2.89 | 2.94 | 3.05 | 2.96 |
| **Conscientiousness** | 2.96 | 3.08 | 3.17 | 3.09 |
| **Extraversion** | 2.53 | 2.67 | 2.74 | 2.72 |
| **Neuroticism** | 2.54 | 2.32 | 2.40 | 2.43 |
| **Openness** | 2.85 | 2.94 | 2.93 | 2.92 |
| **Agreeableness** | 3.05 | 3.04 | 3.07 | 2.98 |
| **Average Item Standard Deviation** | | | | |
|  | **Original** | **Low-to-High** | **High-to-Low** | **Half-Half** |
|  | **(Likert)** | **(Expanded)** | **(Expanded)** | **(Expanded)** |
| **RSE** | 0.81 | 0.70 | 0.62 | 0.70 |
| **Conscientiousness** | 0.81 | 0.70 | 0.62 | 0.70 |
| **Extraversion** | 0.86 | 0.77 | 0.79 | 0.79 |
| **Neuroticism** | 0.90 | 0.77 | 0.81 | 0.80 |
| **Openness** | 0.85 | 0.79 | 0.79 | 0.79 |
| **Agreeableness** | 0.81 | 0.81 | 0.77 | 0.83 |

Note: Scores range from 1 to 4 for all items. Higher values indicate higher endorsement of the construct.
