# Supplementary material for: Improved Properties of the Big Five Inventory and the Rosenberg Self-Esteem Scale in the Expanded Format Relative to the Likert Format
Source: Front Psychol. 2019 Jun 4;10:1286. doi: 10.3389/fpsyg.2019.01286 (PMC6558198; doi:10.3389/fpsyg.2019.01286)
Supplement: Supplementary file 2 [file Table_2.DOCX]

**All Versions of the Rosenberg Self-Esteem Scale and the Big Five Inventory**

**Rosenberg Self-Esteem Scale (Original Version)**

For each of the following, please pick the one that corresponds with the answer that best describes how strongly you agree or disagree with the statement about yourself now.

|  | Strongly disagree | Disagree | Agree | Strongly agree |
| --- | --- | --- | --- | --- |
| I feel that I am a person of worth, or at least on an equal plane with others. |  |  |  |  |
| I feel that I have a number of good qualities. |  |  |  |  |
| All in all, I'm inclined to feel that I am a failure. |  |  |  |  |
| I am able to do things as well as most other people. |  |  |  |  |
| I feel I do not have much to be proud of. |  |  |  |  |
| I take a positive attitude towards myself. |  |  |  |  |
| On the whole, I am satisfied with myself. |  |  |  |  |
| I certainly feel useless at times. |  |  |  |  |
| I wish I could have more respect for myself. |  |  |  |  |
| At times, I think I am no good at all. |  |  |  |  |

Note: Item 3, 5, 8, 9, and 10 are reverse worded items.

**Rosenberg Self-Esteem Scale (Low-to-High Version)**

This questionnaire consists of 10 groups of statements. Please read each group of statements carefully, and then pick out the one statement in each group that best describes you. Be sure that you do not choose more than one statement for any group.

1. Select the one of the following options that best describes you.

- I feel that I’m a person of little worth, less so than other people.
- I feel that I’m a person of little worth, not on an equal basis with others.
- I feel that I’m a person of worth, at least on an equal basis with others.
- I feel that I’m a person of great worth, more so than other people.

1. Select the one of the following options that best describes you.

- I feel that I have very few good qualities.
- I feel that I don’t have many good qualities.
- I feel that I have some good qualities.
- I feel that I have a great number of good qualities.

1. Select the one of the following options that best describes you.

- All in all, I think I am a failure.
- All in all, I think I am somewhat a failure.
- All in all, I think I am somewhat a success.
- All in all, I think I am a success.

1. Select the one of the following options that best describes you.

- I usually do things much worse than other people.
- I am unable to do things as well as most other people.
- I am able to do things as well as most other people.
- I am able to do things much better than most other people.

1. Select the one of the following options that best describes you.

- I feel I have nothing to be proud of.
- I feel I do not have much to be proud of.
- I feel I have some qualities that I feel proud of.
- I feel I have a lot to be proud of.

1. Select the one of the following options that best describes you.

- I take a very negative attitude towards myself.
- I take a negative attitude towards myself.
- I take a positive attitude towards myself.
- I take a very positive attitude towards myself.

1. Select the one of the following options that best describes you.

- On the whole, I am very disappointed with myself.
- On the whole, I am disappointed with myself.
- On the whole, I am satisfied with myself.
- On the whole, I am very satisfied with myself.

1. Select the one of the following options that best describes you.

- I certainly feel useless most of the time.
- I certainly feel useless at times.
- I certainly feel useful at times.
- I certainly feel useful most of the time.

1. Select the one of the following options that best describes you.

- I have no respect for myself.
- I have little respect for myself.
- I have some respect for myself.
- I have a lot of respect for myself.

1. Select the one of the following options that best describes you.

- Most of the time, I think I’m no good at all.
- At times, I think I’m no good at all.
- At times, I think I’m fairly good.
- Most of the time, I think I’m very good.

**Rosenberg Self-Esteem Scale (High-to-Low Version)**

This questionnaire consists of 10 groups of statements. Please read each group of statements carefully, and then pick out the one statement in each group that best describes you. Be sure that you do not choose more than one statement for any group.

1. Select the one of the following options that best describes you.

- I feel that I’m a person of great worth, more so than other people.
- I feel that I’m a person of worth, at least on an equal basis with others.
- I feel that I’m a person of little worth, not on an equal basis with others.
- I feel that I’m a person of little worth, less so than other people.

1. Select the one of the following options that best describes you.

- I feel that I have a great number of good qualities.
- I feel that I have some good qualities.
- I feel that I don’t have many good qualities.
- I feel that I have very few good qualities.

1. Select the one of the following options that best describes you.

- All in all, I think I am a success.
- All in all, I think I am somewhat a success.
- All in all, I think I am somewhat a failure.
- All in all, I think I am a failure.

1. Select the one of the following options that best describes you.

- I am able to do things much better than most other people.
- I am able to do things as well as most other people.
- I am unable to do things as well as most other people.
- I usually do things much worse than other people.

1. Select the one of the following options that best describes you.

- I feel I have a lot to be proud of.
- I feel I have some qualities that I feel proud of.
- I feel I do not have much to be proud of.
- I feel I have nothing to be proud of.

1. Select the one of the following options that best describes you.

- I take a very positive attitude towards myself.
- I take a positive attitude towards myself.
- I take a negative attitude towards myself.
- I take a very negative attitude towards myself.

1. Select the one of the following options that best describes you.

- On the whole, I am very satisfied with myself.
- On the whole, I am satisfied with myself.
- On the whole, I am disappointed with myself.
- On the whole, I am very disappointed with myself.

1. Select the one of the following options that best describes you.

- I certainly feel useful most of the time.
- I certainly feel useful at times.
- I certainly feel useless at times.
- I certainly feel useless most of the time.

1. Select the one of the following options that best describes you.

- I have a lot of respect for myself.
- I have some respect for myself.
- I have little respect for myself.
- I have no respect for myself.

1. Select the one of the following options that best describes you.

- Most of the time, I think I’m very good.
- At times, I think I’m fairly good.
- At times, I think I’m no good at all.
- Most of the time, I think I’m no good at all.

**Rosenberg Self-Esteem Scale (Half-Half Version)**

This questionnaire consists of 10 groups of statements. Please read each group of statements carefully, and then pick out the one statement in each group that best describes you. Be sure that you do not choose more than one statement for any group.

1. Select the one of the following options that best describes you.

- I feel that I’m a person of little worth, less so than other people.
- I feel that I’m a person of little worth, not on an equal basis with others.
- I feel that I’m a person of worth, at least on an equal basis with others.
- I feel that I’m a person of great worth, more so than other people.

1. Select the one of the following options that best describes you.

- I feel that I have very few good qualities.
- I feel that I don’t have many good qualities.
- I feel that I have some good qualities.
- I feel that I have a great number of good qualities.

1. Select the one of the following options that best describes you.

- All in all, I think I am a success.
- All in all, I think I am somewhat a success.
- All in all, I think I am somewhat a failure.
- All in all, I think I am a failure.

1. Select the one of the following options that best describes you.

- I usually do things much worse than other people.
- I am unable to do things as well as most other people.
- I am able to do things as well as most other people.
- I am able to do things much better than most other people.

1. Select the one of the following options that best describes you.

- I feel I have a lot to be proud of.
- I feel I have some qualities that I feel proud of.
- I feel I do not have much to be proud of.
- I feel I have nothing to be proud of.

1. Select the one of the following options that best describes you.

- I take a very negative attitude towards myself.
- I take a negative attitude towards myself.
- I take a positive attitude towards myself.
- I take a very positive attitude towards myself.

1. Select the one of the following options that best describes you.

- On the whole, I am very disappointed with myself.
- On the whole, I am disappointed with myself.
- On the whole, I am satisfied with myself.
- On the whole, I am very satisfied with myself.

1. Select the one of the following options that best describes you.

- I certainly feel useful most of the time.
- I certainly feel useful at times.
- I certainly feel useless at times.
- I certainly feel useless most of the time.

1. Select the one of the following options that best describes you.

- I have a lot of respect for myself.
- I have some respect for myself.
- I have little respect for myself.
- I have no respect for myself.

1. Select the one of the following options that best describes you.

- Most of the time, I think I’m very good.
- At times, I think I’m fairly good.
- At times, I think I’m no good at all.
- Most of the time, I think I’m no good at all.

**Conscientiousness Scale (Original Version)**

Here are a number of characteristics that may or may not apply to you. Please rate the extent to which you agree or disagree with that statement.

|  | Disagree strongly | Disagree a little | Agree a little | Agree strongly |
| --- | --- | --- | --- | --- |
| I am someone who does a thorough job. |  |  |  |  |
| I am someone who can be somewhat careless. |  |  |  |  |
| I am someone who is a reliable worker. |  |  |  |  |
| I am someone who tends to be disorganized. |  |  |  |  |
| I am someone who tends to be lazy. |  |  |  |  |
| I am someone who perseveres until the task is finished. |  |  |  |  |
| I am someone who does things efficiently. |  |  |  |  |
| I am someone who makes plans and follows through on them. |  |  |  |  |
| I am someone who is easily distracted. |  |  |  |  |

Note: Item 2, 4, 5, and 9 are reverse worded items.

**Conscientiousness Scale (Low-to-High Version)**

This questionnaire consists of 9 groups of statements. Please read each group of statements carefully, and then pick out the one statement in each group that best describes you. Be sure that you do not choose more than one statement for any group.

1. Select the one of the following options that best describes you.

- I am someone who does a very sloppy job.
- I am someone who does a somewhat sloppy job.
- I am someone who does a somewhat thorough job.
- I am someone who does a very thorough job.

1. Select the one of the following options that best describes you.

- I am someone who can be very careless.
- I am someone who can be somewhat careless.
- I am someone who can be somewhat careful.
- I am someone who can be very careful.

1. Select the one of the following options that best describes you.

- I am someone who is a very unreliable worker.
- I am someone who is a somewhat unreliable worker.
- I am someone who is a somewhat reliable worker.
- I am someone who is a very reliable worker.

1. Select the one of the following options that best describes you.

- I am someone who tends to be very disorganized.
- I am someone who tends to be somewhat disorganized.
- I am someone who tends to be somewhat organized.
- I am someone who tends to be very organized.

1. Select the one of the following options that best describes you.

- I am someone who tends to be very lazy.
- I am someone who tends to be somewhat lazy.
- I am someone who tends to be somewhat diligent.
- I am someone who tends to be very diligent.

1. Select the one of the following options that best describes you.

- I am someone who often gives up before the task is finished.
- I am someone who gives up before the task is finished.
- I am someone who perseveres until the task is finished.
- I am someone who often perseveres until the task is finished.

1. Select the one of the following options that best describes you.

- I am someone who does things very inefficiently.
- I am someone who does things somewhat inefficiently.
- I am someone who does things somewhat efficiently.
- I am someone who does things very efficiently.

1. Select the one of the following options that best describes you.

- I am someone who makes plans but rarely follows through on them.
- I am someone who makes plans but does not usually follow through on them.
- I am someone who makes plans and usually follows through on them.
- I am someone who makes plans and always follows through on them.

1. Select the one of the following options that best describes you.

- I am someone who is very easily distracted.
- I am someone who is easily distracted.
- I am someone who can easily stay focused.
- I am someone who can very easily stay focused.

**Conscientiousness Scale (High-to-Low Version)**

This questionnaire consists of 9 groups of statements. Please read each group of statements carefully, and then pick out the one statement in each group that best describes you. Be sure that you do not choose more than one statement for any group.

1. Select the one of the following options that best describes you.

- I am someone who does a very thorough job.
- I am someone who does a somewhat thorough job.
- I am someone who does a somewhat sloppy job.
- I am someone who does a very sloppy job.

1. Select the one of the following options that best describes you.

- I am someone who can be very careful.
- I am someone who can be somewhat careful.
- I am someone who can be somewhat careless.
- I am someone who can be very careless.

1. Select the one of the following options that best describes you.

- I am someone who is a very reliable worker.
- I am someone who is a somewhat reliable worker.
- I am someone who is a somewhat unreliable worker.
- I am someone who is a very unreliable worker.

1. Select the one of the following options that best describes you.

- I am someone who tends to be very organized.
- I am someone who tends to be somewhat organized.
- I am someone who tends to be somewhat disorganized.
- I am someone who tends to be very disorganized.

1. Select the one of the following options that best describes you.

- I am someone who tends to be very diligent.
- I am someone who tends to be somewhat diligent.
- I am someone who tends to be somewhat lazy.
- I am someone who tends to be very lazy.

1. Select the one of the following options that best describes you.

- I am someone who often perseveres until the task is finished.
- I am someone who perseveres until the task is finished.
- I am someone who gives up before the task is finished.
- I am someone who often gives up before the task is finished.

1. Select the one of the following options that best describes you.

- I am someone who does things very efficiently.
- I am someone who does things somewhat efficiently.
- I am someone who does things somewhat inefficiently.
- I am someone who does things very inefficiently.

1. Select the one of the following options that best describes you.

- I am someone who makes plans and always follows through on them.
- I am someone who makes plans and usually follows through on them.
- I am someone who makes plans but does not usually follow through on them.
- I am someone who makes plans but rarely follows through on them.

1. Select the one of the following options that best describes you.

- I am someone who can very easily stay focused.
- I am someone who can easily stay focused.
- I am someone who is easily distracted.
- I am someone who is very easily distracted.

**Conscientiousness Scale (Half-Half Version)**

This questionnaire consists of 9 groups of statements. Please read each group of statements carefully, and then pick out the one statement in each group that best describes you. Be sure that you do not choose more than one statement for any group.

1. Select the one of the following options that best describes you.

- I am someone who does a very sloppy job.
- I am someone who does a somewhat sloppy job.
- I am someone who does a somewhat thorough job.
- I am someone who does a very thorough job.

1. Select the one of the following options that best describes you.

- I am someone who can be very careful.
- I am someone who can be somewhat careful.
- I am someone who can be somewhat careless.
- I am someone who can be very careless.

1. Select the one of the following options that best describes you.

- I am someone who is a very unreliable worker.
- I am someone who is a somewhat unreliable worker.
- I am someone who is a somewhat reliable worker.
- I am someone who is a very reliable worker.

1. Select the one of the following options that best describes you.

- I am someone who tends to be very organized.
- I am someone who tends to be somewhat organized.
- I am someone who tends to be somewhat disorganized.
- I am someone who tends to be very disorganized.

1. Select the one of the following options that best describes you.

- I am someone who tends to be very diligent.
- I am someone who tends to be somewhat diligent.
- I am someone who tends to be somewhat lazy.
- I am someone who tends to be very lazy.

1. Select the one of the following options that best describes you.

- I am someone who often gives up before the task is finished.
- I am someone who gives up before the task is finished.
- I am someone who perseveres until the task is finished.
- I am someone who often perseveres until the task is finished.

1. Select the one of the following options that best describes you.

- I am someone who does things very inefficiently.
- I am someone who does things somewhat inefficiently.
- I am someone who does things somewhat efficiently.
- I am someone who does things very efficiently.

1. Select the one of the following options that best describes you.

- I am someone who makes plans but rarely follows through on them.
- I am someone who makes plans but does not usually follow through on them.
- I am someone who makes plans and usually follows through on them.
- I am someone who makes plans and always follows through on them.

1. Select the one of the following options that best describes you.

- I am someone who can very easily stay focused.
- I am someone who can easily stay focused.
- I am someone who is easily distracted.
- I am someone who is very easily distracted.

**Extraversion Scale (Original Version)**

Here are a number of characteristics that may or may not apply to you. Please rate the extent to which you agree or disagree with that statement.

|  | Disagree strongly | Disagree a little | Agree a little | Agree strongly |
| --- | --- | --- | --- | --- |
| I am someone who is talkative. |  |  |  |  |
| I am someone who is reserved. |  |  |  |  |
| I am someone who is full of energy. |  |  |  |  |
| I am someone who generates a lot of enthusiasm. |  |  |  |  |
| I am someone who tends to be quiet. |  |  |  |  |
| I am someone who has an assertive personality. |  |  |  |  |
| I am someone who is sometimes shy, inhibited. |  |  |  |  |
| I am someone who is outgoing, sociable. |  |  |  |  |

Note: Item 2, 5, and 7 are reverse worded items.

**Extraversion Scale (Low-to-High Version)**

This questionnaire consists of 8 groups of statements. Please read each group of statements carefully, and then pick out the one statement in each group that best describes you. Be sure that you do not choose more than one statement for any group.

1. Select the one of the following options that best describes you.

- I am someone who is very untalkative.
- I am someone who is somewhat untalkative.
- I am someone who is somewhat talkative.
- I am someone who is very talkative.

1. Select the one of the following options that best describes you.

- I am someone who is very reserved.
- I am someone who is somewhat reserved.
- I am someone who is somewhat open.
- I am someone who is very open.

1. Select the one of the following options that best describes you.

- I am someone who almost always lacks energy.
- I am someone who often lacks energy.
- I am someone who is often full of energy.
- I am someone who is almost always full of energy.

1. Select the one of the following options that best describes you.

- I am someone who generates almost no enthusiasm.
- I am someone who generates a little enthusiasm.
- I am someone who generates some enthusiasm.
- I am someone who generates a lot of enthusiasm.

1. Select the one of the following options that best describes you.

- I am someone who tends to be very quiet.
- I am someone who tends to be somewhat quiet.
- I am someone who tends to be somewhat loud.
- I am someone who tends to be very loud.

1. Select the one of the following options that best describes you.

- I am someone who has a very submissive personality.
- I am someone who has a somewhat submissive personality.
- I am someone who has a somewhat assertive personality.
- I am someone who has a very assertive personality.

1. Select the one of the following options that best describes you.

- I am someone who is almost always shy and inhibited.
- I am someone who is often shy and inhibited.
- I am someone who is sometimes shy and inhibited.
- I am someone who is rarely shy and inhibited.

1. Select the one of the following options that best describes you.

- I am someone who is very reclusive and unsociable.
- I am someone who is somewhat reclusive and unsociable.
- I am someone who is somewhat outgoing and sociable.
- I am someone who is very outgoing and sociable.

**Extraversion Scale (High-to-Low Version)**

This questionnaire consists of 8 groups of statements. Please read each group of statements carefully, and then pick out the one statement in each group that best describes you. Be sure that you do not choose more than one statement for any group.

1. Select the one of the following options that best describes you.

- I am someone who is very talkative.
- I am someone who is somewhat talkative.
- I am someone who is somewhat untalkative.
- I am someone who is very untalkative.

1. Select the one of the following options that best describes you.

- I am someone who is very open.
- I am someone who is somewhat open.
- I am someone who is somewhat reserved.
- I am someone who is very reserved.

1. Select the one of the following options that best describes you.

- I am someone who is almost always full of energy.
- I am someone who is often full of energy.
- I am someone who often lacks energy.
- I am someone who almost always lacks energy.

1. Select the one of the following options that best describes you.

- I am someone who generates a lot of enthusiasm.
- I am someone who generates some enthusiasm.
- I am someone who generates a little enthusiasm.
- I am someone who generates almost no enthusiasm.

1. Select the one of the following options that best describes you.

- I am someone who tends to be very loud.
- I am someone who tends to be somewhat loud.
- I am someone who tends to be somewhat quiet.
- I am someone who tends to be very quiet.

1. Select the one of the following options that best describes you.

- I am someone who has a very assertive personality.
- I am someone who has a somewhat assertive personality.
- I am someone who has a somewhat submissive personality.
- I am someone who has a very submissive personality.

1. Select the one of the following options that best describes you.

- I am someone who is rarely shy and inhibited.
- I am someone who is sometimes shy and inhibited.
- I am someone who is often shy and inhibited.
- I am someone who is almost always shy and inhibited.

1. Select the one of the following options that best describes you.

- I am someone who is very outgoing and sociable.
- I am someone who is somewhat outgoing and sociable.
- I am someone who is somewhat reclusive and unsociable.
- I am someone who is very reclusive and unsociable.

**Extraversion Scale (Half-Half Version)**

This questionnaire consists of 8 groups of statements. Please read each group of statements carefully, and then pick out the one statement in each group that best describes you. Be sure that you do not choose more than one statement for any group.

1. Select the one of the following options that best describes you.

- I am someone who is very untalkative.
- I am someone who is somewhat untalkative.
- I am someone who is somewhat talkative.
- I am someone who is very talkative.

1. Select the one of the following options that best describes you.

- I am someone who is very open.
- I am someone who is somewhat open.
- I am someone who is somewhat reserved.
- I am someone who is very reserved.

1. Select the one of the following options that best describes you.

- I am someone who almost always lacks energy.
- I am someone who often lacks energy.
- I am someone who is often full of energy.
- I am someone who is almost always full of energy.

1. Select the one of the following options that best describes you.

- I am someone who generates almost no enthusiasm.
- I am someone who generates a little enthusiasm.
- I am someone who generates some enthusiasm.
- I am someone who generates a lot of enthusiasm.

1. Select the one of the following options that best describes you.

- I am someone who tends to be very loud.
- I am someone who tends to be somewhat loud.
- I am someone who tends to be somewhat quiet.
- I am someone who tends to be very quiet.

1. Select the one of the following options that best describes you.

- I am someone who has a very submissive personality.
- I am someone who has a somewhat submissive personality.
- I am someone who has a somewhat assertive personality.
- I am someone who has a very assertive personality.

1. Select the one of the following options that best describes you.

- I am someone who is rarely shy and inhibited.
- I am someone who is sometimes shy and inhibited.
- I am someone who is often shy and inhibited.
- I am someone who is almost always shy and inhibited.

1. Select the one of the following options that best describes you.

- I am someone who is very reclusive and unsociable.
- I am someone who is somewhat reclusive and unsociable.
- I am someone who is somewhat outgoing and sociable.
- I am someone who is very outgoing and sociable.

**Neuroticism Scale (Original Version)**

Here are a number of characteristics that may or may not apply to you. Please rate the extent to which you agree or disagree with that statement.

|  | Disagree strongly | Disagree a little | Agree a little | Agree strongly |
| --- | --- | --- | --- | --- |
| I am someone who is depressed, blue. |  |  |  |  |
| I am someone who is relaxed, handles stress well. |  |  |  |  |
| I am someone who worries a lot. |  |  |  |  |
| I am someone who can be tense. |  |  |  |  |
| I am someone who is emotionally stable, not easily upset. |  |  |  |  |
| I am someone who can be moody. |  |  |  |  |
| I am someone who remains calm in tense situations. |  |  |  |  |
| I am someone who gets nervous easily. |  |  |  |  |

Note: Item 2, 5, and 7 are reverse worded items.

**Neuroticism Scale (Low-to-High Version)**

This questionnaire consists of 8 groups of statements. Please read each group of statements carefully, and then pick out the one statement in each group that best describes you. Be sure that you do not choose more than one statement for any group.

1. Select the one of the following options that best describes you.

- I am someone who is very cheerful and happy.
- I am someone who is somewhat cheerful and happy.
- I am someone who is somewhat depressed and blue.
- I am someone who is very depressed and blue.

1. Select the one of the following options that best describes you.

- I am someone who is completely relaxed and handles stress very well.
- I am someone who is fairly relaxed and handles stress well.
- I am someone who is not very relaxed and doesn’t handle stress very well.
- I am someone who is not at all relaxed and handles stress poorly.

1. Select the one of the following options that best describes you.

- I am someone who rarely worries.
- I am someone who sometimes worries.
- I am someone who often worries.
- I am someone who almost always worries.

1. Select the one of the following options that best describes you.

- I am someone who is rarely tense.
- I am someone who is sometimes tense.
- I am someone who is often tense.
- I am someone who is almost always tense.

1. Select the one of the following options that best describes you.

- I am someone who is very emotionally stable and rarely gets upset.
- I am someone who is emotionally stable and does not often get upset.
- I am someone who is emotionally unstable and gets upset sometimes.
- I am someone who is very emotionally unstable and gets upset often.

1. Select the one of the following options that best describes you.

- I am someone who is rarely moody.
- I am someone who is sometimes moody.
- I am someone who is often moody.
- I am someone who is almost always moody.

1. Select the one of the following options that best describes you.

- I am someone who remains very calm in tense situations.
- I am someone who remains calm in tense situations.
- I am someone who gets panicky in tense situations.
- I am someone who gets very panicky in tense situations.

1. Select the one of the following options that best describes you.

- I am someone who hardly gets nervous.
- I am someone who doesn’t get nervous easily.
- I am someone who gets nervous easily.
- I am someone who gets nervous very easily.

**Neuroticism Scale (High-to-Low Version)**

This questionnaire consists of 8 groups of statements. Please read each group of statements carefully, and then pick out the one statement in each group that best describes you. Be sure that you do not choose more than one statement for any group.

1. Select the one of the following options that best describes you.

- I am someone who is very depressed and blue.
- I am someone who is somewhat depressed and blue.
- I am someone who is somewhat cheerful and happy.
- I am someone who is very cheerful and happy.

1. Select the one of the following options that best describes you.

- I am someone who is not at all relaxed and handles stress poorly.
- I am someone who is not very relaxed and doesn’t handle stress very well.
- I am someone who is fairly relaxed and handles stress well.
- I am someone who is completely relaxed and handles stress very well.

1. Select the one of the following options that best describes you.

- I am someone who almost always worries.
- I am someone who often worries.
- I am someone who sometimes worries.
- I am someone who rarely worries.

1. Select the one of the following options that best describes you.

- I am someone who is almost always tense.
- I am someone who is often tense.
- I am someone who is sometimes tense.
- I am someone who is rarely tense.

1. Select the one of the following options that best describes you.

- I am someone who is very emotionally unstable and gets upset often.
- I am someone who is emotionally unstable and gets upset sometimes.
- I am someone who is emotionally stable and does not often get upset.
- I am someone who is very emotionally stable and rarely gets upset.

1. Select the one of the following options that best describes you.

- I am someone who is almost always moody.
- I am someone who is often moody.
- I am someone who is sometimes moody.
- I am someone who is rarely moody.

1. Select the one of the following options that best describes you.

- I am someone who gets very panicky in tense situations.
- I am someone who gets panicky in tense situations.
- I am someone who remains calm in tense situations.
- I am someone who remains very calm in tense situations.

1. Select the one of the following options that best describes you.

- I am someone who gets nervous very easily.
- I am someone who gets nervous easily.
- I am someone who doesn’t get nervous easily.
- I am someone who hardly gets nervous.

**Neuroticism Scale (Half-Half Version)**

This questionnaire consists of 8 groups of statements. Please read each group of statements carefully, and then pick out the one statement in each group that best describes you. Be sure that you do not choose more than one statement for any group.

1. Select the one of the following options that best describes you.

- I am someone who is very cheerful and happy.
- I am someone who is somewhat cheerful and happy.
- I am someone who is somewhat depressed and blue.
- I am someone who is very depressed and blue.

1. Select the one of the following options that best describes you.

- I am someone who is not at all relaxed and handles stress poorly.
- I am someone who is not very relaxed and doesn’t handle stress very well.
- I am someone who is fairly relaxed and handles stress well.
- I am someone who is completely relaxed and handles stress very well.

1. Select the one of the following options that best describes you.

- I am someone who rarely worries.
- I am someone who sometimes worries.
- I am someone who often worries.
- I am someone who almost always worries.

1. Select the one of the following options that best describes you.

- I am someone who is rarely tense.
- I am someone who is sometimes tense.
- I am someone who is often tense.
- I am someone who is almost always tense.

1. Select the one of the following options that best describes you.

- I am someone who is very emotionally unstable and gets upset often.
- I am someone who is emotionally unstable and gets upset sometimes.
- I am someone who is emotionally stable and does not often get upset.
- I am someone who is very emotionally stable and rarely gets upset.

1. Select the one of the following options that best describes you.

- I am someone who is rarely moody.
- I am someone who is sometimes moody.
- I am someone who is often moody.
- I am someone who is almost always moody.

1. Select the one of the following options that best describes you.

- I am someone who gets very panicky in tense situations.
- I am someone who gets panicky in tense situations.
- I am someone who remains calm in tense situations.
- I am someone who remains very calm in tense situations.

1. Select the one of the following options that best describes you.

- I am someone who hardly gets nervous.
- I am someone who doesn’t get nervous easily.
- I am someone who gets nervous easily.
- I am someone who gets nervous very easily.

**Openness Scale (Original Version)**

Here are a number of characteristics that may or may not apply to you. Please rate the extent to which you agree or disagree with that statement.

|  | Disagree strongly | Disagree a little | Agree a little | Agree strongly |
| --- | --- | --- | --- | --- |
| I am someone who is original, comes up with new ideas. |  |  |  |  |
| I am someone who has an active imagination. |  |  |  |  |
| I am someone who is inventive. |  |  |  |  |
| I am someone who values artistic, aesthetic experiences. |  |  |  |  |
| I am someone who prefers work that is routine. |  |  |  |  |
| I am someone who is ingenious, a deep thinker. |  |  |  |  |
| I am someone who likes to reflect, play with ideas. |  |  |  |  |
| I am someone who few artistic interests. |  |  |  |  |
| I am someone who is sophisticated in art, music or literature. |  |  |  |  |
| I am someone who is curious about many different things. |  |  |  |  |

Note: Item 5 and 7 are reverse worded items.

**Openness Scale (Low-to-High Version)**

This questionnaire consists of 10 groups of statements. Please read each group of statements carefully, and then pick out the one statement in each group that best describes you. Be sure that you do not choose more than one statement for any group.

1. Select the one of the following options that best describes you.

- I am someone who is unoriginal at all and very rarely comes up with new ideas.
- I am someone who is somewhat unoriginal and rarely comes up with new ideas.
- I am someone who is somewhat original and occasionally comes up with new ideas.
- I am someone who is original and often comes up with new ideas.

1. Select the one of the following options that best describes you.

- I am someone who is very unimaginative.
- I am someone who is somewhat unimaginative.
- I am someone who is somewhat imaginative.
- I am someone who is very imaginative.

1. Select the one of the following options that best describes you.

- I am someone who is very uninventive.
- I am someone who is somewhat uninventive.
- I am someone who is somewhat inventive.
- I am someone who is very inventive.

1. Select the one of the following options that best describes you.

- I am someone who doesn’t value artistic and aesthetic experiences at all.
- I am someone who doesn’t really value artistic and aesthetic experiences.
- I am someone who somewhat values artistic and aesthetic experiences.
- I am someone who really values artistic and aesthetic experiences.

1. Select the one of the following options that best describes you.

- I am someone who really prefers work that is routine.
- I am someone who prefers work that is routine.
- I am someone who hates work that is routine.
- I am someone who really hates work that is routine.

1. Select the one of the following options that best describes you.

- I am someone who is very uncreative and is not at all a deep thinker.
- I am someone who is somewhat uncreative and is not a very deep thinker.
- I am someone who is somewhat ingenious and is a somewhat deep thinker.
- I am someone who is very ingenious and is a very deep thinker.

1. Select the one of the following options that best describes you.

- I am someone who really dislikes to reflect and to play with ideas.
- I am someone who dislikes to reflect and to play with ideas.
- I am someone who likes to reflect and to play with ideas.
- I am someone who really likes to reflect and to play with ideas.

1. Select the one of the following options that best describes you.

- I am someone who has very few artistic interests.
- I am someone who has few artistic interests.
- I am someone who has some artistic interests.
- I am someone who has many artistic interests.

1. Select the one of the following options that best describes you.

- I am someone who is very unsophisticated in art, music or literature.
- I am someone who is somewhat unsophisticated in art, music or literature.
- I am someone who is somewhat sophisticated in art, music or literature.
- I am someone who is very sophisticated in art, music or literature.

1. Select the one of the following options that best describes you.

- I am someone who is curious about very few different things.
- I am someone who is curious about few different things.
- I am someone who is curious about quite a few different things.
- I am someone who is curious about a lot of different things.

**Openness Scale (High-to-Low Version)**

This questionnaire consists of 10 groups of statements. Please read each group of statements carefully, and then pick out the one statement in each group that best describes you. Be sure that you do not choose more than one statement for any group.

1. Select the one of the following options that best describes you.

- I am someone who is original and often comes up with new ideas.
- I am someone who is somewhat original and occasionally comes up with new ideas.
- I am someone who is somewhat unoriginal and rarely comes up with new ideas.
- I am someone who is unoriginal at all and very rarely comes up with new ideas.

1. Select the one of the following options that best describes you.

- I am someone who is very imaginative.
- I am someone who is somewhat imaginative.
- I am someone who is somewhat unimaginative.
- I am someone who is very unimaginative.

1. Select the one of the following options that best describes you.

- I am someone who is very inventive.
- I am someone who is somewhat inventive.
- I am someone who is somewhat uninventive.
- I am someone who is very uninventive.

1. Select the one of the following options that best describes you.

- I am someone who really values artistic and aesthetic experiences.
- I am someone who somewhat values artistic and aesthetic experiences.
- I am someone who doesn’t really value artistic and aesthetic experiences.
- I am someone who doesn’t value artistic and aesthetic experiences at all.

1. Select the one of the following options that best describes you.

- I am someone who really hates work that is routine.
- I am someone who hates work that is routine.
- I am someone who prefers work that is routine.
- I am someone who really prefers work that is routine.

1. Select the one of the following options that best describes you.

- I am someone who is very ingenious and is a very deep thinker.
- I am someone who is somewhat ingenious and is a somewhat deep thinker.
- I am someone who is somewhat uncreative and is not a very deep thinker.
- I am someone who is very uncreative and is not at all a deep thinker.

1. Select the one of the following options that best describes you.

- I am someone who really likes to reflect and to play with ideas.
- I am someone who likes to reflect and to play with ideas.
- I am someone who dislikes to reflect and to play with ideas.
- I am someone who really dislikes to reflect and to play with ideas.

1. Select the one of the following options that best describes you.

- I am someone who has many artistic interests.
- I am someone who has some artistic interests.
- I am someone who has few artistic interests.
- I am someone who has very few artistic interests.

1. Select the one of the following options that best describes you.

- I am someone who is very sophisticated in art, music or literature.
- I am someone who is somewhat sophisticated in art, music or literature.
- I am someone who is somewhat unsophisticated in art, music or literature.
- I am someone who is very unsophisticated in art, music or literature.

1. Select the one of the following options that best describes you.

- I am someone who is curious about a lot of different things.
- I am someone who is curious about quite a few different things.
- I am someone who is curious about few different things.
- I am someone who is curious about very few different things.

**Openness Scale (Half-Half Version)**

This questionnaire consists of 10 groups of statements. Please read each group of statements carefully, and then pick out the one statement in each group that best describes you. Be sure that you do not choose more than one statement for any group.

1. Select the one of the following options that best describes you.

- I am someone who is unoriginal at all and very rarely comes up with new ideas.
- I am someone who is somewhat unoriginal and rarely comes up with new ideas.
- I am someone who is somewhat original and occasionally comes up with new ideas.
- I am someone who is original and often comes up with new ideas.

1. Select the one of the following options that best describes you.

- I am someone who is very unimaginative.
- I am someone who is somewhat unimaginative.
- I am someone who is somewhat imaginative.
- I am someone who is very imaginative.

1. Select the one of the following options that best describes you.

- I am someone who is very uninventive.
- I am someone who is somewhat uninventive.
- I am someone who is somewhat inventive.
- I am someone who is very inventive.

1. Select the one of the following options that best describes you.

- I am someone who doesn’t value artistic and aesthetic experiences at all.
- I am someone who doesn’t really value artistic and aesthetic experiences.
- I am someone who somewhat values artistic and aesthetic experiences.
- I am someone who really values artistic and aesthetic experiences.

1. Select the one of the following options that best describes you.

- I am someone who really hates work that is routine.
- I am someone who hates work that is routine.
- I am someone who prefers work that is routine.
- I am someone who really prefers work that is routine.

1. Select the one of the following options that best describes you.

- I am someone who is very uncreative and is not at all a deep thinker.
- I am someone who is somewhat uncreative and is not a very deep thinker.
- I am someone who is somewhat ingenious and is a somewhat deep thinker.
- I am someone who is very ingenious and is a very deep thinker.

1. Select the one of the following options that best describes you.

- I am someone who really dislikes to reflect and to play with ideas.
- I am someone who dislikes to reflect and to play with ideas.
- I am someone who likes to reflect and to play with ideas.
- I am someone who really likes to reflect and to play with ideas.

1. Select the one of the following options that best describes you.

- I am someone who has many artistic interests.
- I am someone who has some artistic interests.
- I am someone who has few artistic interests.
- I am someone who has very few artistic interests.

1. Select the one of the following options that best describes you.

- I am someone who is very unsophisticated in art, music or literature.
- I am someone who is somewhat unsophisticated in art, music or literature.
- I am someone who is somewhat sophisticated in art, music or literature.
- I am someone who is very sophisticated in art, music or literature.

1. Select the one of the following options that best describes you.

- I am someone who is curious about very few different things.
- I am someone who is curious about few different things.
- I am someone who is curious about quite a few different things.
- I am someone who is curious about a lot of different things.

**Agreeableness Scale (Original Version)**

Here are a number of characteristics that may or may not apply to you. Please rate the extent to which you agree or disagree with that statement.

|  | Disagree strongly | Disagree a little | Agree a little | Agree strongly |
| --- | --- | --- | --- | --- |
| I am someone who tends to find fault with others. |  |  |  |  |
| I am someone who is helpful and unselfish with others. |  |  |  |  |
| I am someone who starts quarrels with others. |  |  |  |  |
| I am someone who has a forgiving nature. |  |  |  |  |
| I am someone who is generally trusting. |  |  |  |  |
| I am someone who can be cold and aloof. |  |  |  |  |
| I am someone who is considerate and kind to almost everyone. |  |  |  |  |
| I am someone who is sometimes rude to others. |  |  |  |  |
| I am someone who likes to cooperate with others. |  |  |  |  |

Note: Item 1, 3, 6, and 8 are reverse worded items.

**Agreeableness Scale (Low-to-High Version)**

This questionnaire consists of 9 groups of statements. Please read each group of statements carefully, and then pick out the one statement in each group that best describes you. Be sure that you do not choose more than one statement for any group.

1. Select the one of the following options that best describes you.

- I am someone who often finds fault with others.
- I am someone who sometimes finds fault with others.
- I am someone who rarely finds fault with others.
- I am someone who very rarely finds fault with others.

1. Select the one of the following options that best describes you.

- I am someone who is unhelpful and selfish with others.
- I am someone who is somewhat unhelpful and selfish with others.
- I am someone who is somewhat helpful and unselfish with others.
- I am someone who is helpful and unselfish with others.

1. Select the one of the following options that best describes you.

- I am someone who often starts quarrels with others.
- I am someone who sometimes starts quarrels with others.
- I am someone who rarely starts quarrels with others.
- I am someone who very rarely starts quarrels with others.

1. Select the one of the following options that best describes you.

- I am someone who has an unforgiving nature.
- I am someone who has a somewhat unforgiving nature.
- I am someone who has a somewhat forgiving nature.
- I am someone who has a forgiving nature.

1. Select the one of the following options that best describes you.

- I am someone who is almost always suspicious.
- I am someone who is often suspicious.
- I am someone who is often trusting.
- I am someone who is almost always trusting.

1. Select the one of the following options that best describes you.

- I am someone who can often be cold and aloof.
- I am someone who can sometimes be cold and aloof.
- I am someone who can rarely be cold and aloof.
- I am someone who can very rarely be cold and aloof.

1. Select the one of the following options that best describes you.

- I am someone who is inconsiderate and unkind to almost everyone.
- I am someone who is somewhat inconsiderate and unkind to almost everyone.
- I am someone who is somewhat considerate and kind to almost everyone.
- I am someone who is considerate and kind to almost everyone.

1. Select the one of the following options that best describes you.

- I am someone who is often rude to others.
- I am someone who is sometimes rude to others.
- I am someone who is rarely rude to others.
- I am someone who is very rarely rude to others.

1. Select the one of the following options that best describes you.

- I am someone who really doesn’t like to cooperate with others.
- I am someone who doesn’t like to cooperate with others.
- I am someone who likes to cooperate with others.
- I am someone who really likes to cooperate with others.

**Agreeableness Scale (High-to-Low Version)**

This questionnaire consists of 9 groups of statements. Please read each group of statements carefully, and then pick out the one statement in each group that best describes you. Be sure that you do not choose more than one statement for any group.

1. Select the one of the following options that best describes you.

- I am someone who very rarely finds fault with others.
- I am someone who rarely finds fault with others.
- I am someone who sometimes finds fault with others.
- I am someone who often finds fault with others.

1. Select the one of the following options that best describes you.

- I am someone who is helpful and unselfish with others.
- I am someone who is somewhat helpful and unselfish with others.
- I am someone who is somewhat unhelpful and selfish with others.
- I am someone who is unhelpful and selfish with others.

1. Select the one of the following options that best describes you.

- I am someone who very rarely starts quarrels with others.
- I am someone who rarely starts quarrels with others.
- I am someone who sometimes starts quarrels with others.
- I am someone who often starts quarrels with others.

1. Select the one of the following options that best describes you.

- I am someone who has a forgiving nature.
- I am someone who has a somewhat forgiving nature.
- I am someone who has a somewhat unforgiving nature.
- I am someone who has an unforgiving nature.

1. Select the one of the following options that best describes you.

- I am someone who is almost always trusting.
- I am someone who is often trusting.
- I am someone who is often suspicious.
- I am someone who is almost always suspicious.

1. Select the one of the following options that best describes you.

- I am someone who can very rarely be cold and aloof.
- I am someone who can rarely be cold and aloof.
- I am someone who can sometimes be cold and aloof.
- I am someone who can often be cold and aloof.

1. Select the one of the following options that best describes you.

- I am someone who is considerate and kind to almost everyone.
- I am someone who is somewhat considerate and kind to almost everyone.
- I am someone who is somewhat inconsiderate and unkind to almost everyone.
- I am someone who is inconsiderate and unkind to almost everyone.

1. Select the one of the following options that best describes you.

- I am someone who is very rarely rude to others.
- I am someone who is rarely rude to others.
- I am someone who is sometimes rude to others.
- I am someone who is often rude to others.

1. Select the one of the following options that best describes you.

- I am someone who really likes to cooperate with others.
- I am someone who likes to cooperate with others.
- I am someone who doesn’t like to cooperate with others.
- I am someone who really doesn’t like to cooperate with others.

**Agreeableness Scale (Half-Half Version)**

This questionnaire consists of 9 groups of statements. Please read each group of statements carefully, and then pick out the one statement in each group that best describes you. Be sure that you do not choose more than one statement for any group.

1. Select the one of the following options that best describes you.

- I am someone who very rarely finds fault with others.
- I am someone who rarely finds fault with others.
- I am someone who sometimes finds fault with others.
- I am someone who often finds fault with others.

1. Select the one of the following options that best describes you.

- I am someone who is unhelpful and selfish with others.
- I am someone who is somewhat unhelpful and selfish with others.
- I am someone who is somewhat helpful and unselfish with others.
- I am someone who is helpful and unselfish with others.

1. Select the one of the following options that best describes you.

- I am someone who very rarely starts quarrels with others.
- I am someone who rarely starts quarrels with others.
- I am someone who sometimes starts quarrels with others.
- I am someone who often starts quarrels with others.

1. Select the one of the following options that best describes you.

- I am someone who has an unforgiving nature.
- I am someone who has a somewhat unforgiving nature.
- I am someone who has a somewhat forgiving nature.
- I am someone who has a forgiving nature.

1. Select the one of the following options that best describes you.

- I am someone who is almost always suspicious.
- I am someone who is often suspicious.
- I am someone who is often trusting.
- I am someone who is almost always trusting.

1. Select the one of the following options that best describes you.

- I am someone who can very rarely be cold and aloof.
- I am someone who can rarely be cold and aloof.
- I am someone who can sometimes be cold and aloof.
- I am someone who can often be cold and aloof.

1. Select the one of the following options that best describes you.

- I am someone who is inconsiderate and unkind to almost everyone.
- I am someone who is somewhat inconsiderate and unkind to almost everyone.
- I am someone who is somewhat considerate and kind to almost everyone.
- I am someone who is considerate and kind to almost everyone.

1. Select the one of the following options that best describes you.

- I am someone who is very rarely rude to others.
- I am someone who is rarely rude to others.
- I am someone who is sometimes rude to others.
- I am someone who is often rude to others.

1. Select the one of the following options that best describes you.

- I am someone who really doesn’t like to cooperate with others.
- I am someone who doesn’t like to cooperate with others.
- I am someone who likes to cooperate with others.
- I am someone who really likes to cooperate with others.
