# Supplementary material for: Improved Properties of the Big Five Inventory and the Rosenberg Self-Esteem Scale in the Expanded Format Relative to the Likert Format
Source: Front Psychol. 2019 Jun 4;10:1286. doi: 10.3389/fpsyg.2019.01286 (PMC6558198; doi:10.3389/fpsyg.2019.01286)
Supplement: Supplementary file 3 [file Table_3.DOCX]

**Distributions of the Response Options for All Versions of the Big Five Inventory and the Rosenberg Self-Esteem Scale**

**Table A: Distributions of the Response Options for the Rosenberg Self-Esteem Scale**

|  | **Item 1** | **Item 2** | **Item 3** | **Item 4** | **Item 5** | **Item 6** | **Item 7** | **Item 8** | **Item 9** | **Item 10** |
| --- | --- | --- | --- | --- | --- | --- | --- | --- | --- | --- |
| **Original (Likert)** |  |  |  |  |  |  |  |  |  |  |
| Response Option 1 | 5 | 1 | 8 | 6 | 11 | 10 | 18 | 31 | 36 | 26 |
| Response Option 2 | 25 | 25 | 57 | 39 | 72 | 55 | 66 | 124 | 108 | 98 |
| Response Option 3 | 166 | 165 | 126 | 173 | 138 | 176 | 170 | 97 | 106 | 86 |
| Response Option 4 | 93 | 98 | 98 | 71 | 68 | 48 | 35 | 37 | 39 | 79 |
| **Low-to-High (Expanded)** |  |  |  |  |  |  |  |  |  |  |
| Response Option 1 | 14 | 16 | 6 | 4 | 7 | 15 | 10 | 12 | 3 | 12 |
| Response Option 2 | 37 | 28 | 62 | 34 | 45 | 78 | 68 | 92 | 17 | 56 |
| Response Option 3 | 222 | 170 | 183 | 213 | 172 | 168 | 191 | 143 | 142 | 172 |
| Response Option 4 | 19 | 78 | 41 | 41 | 68 | 31 | 23 | 45 | 130 | 52 |
| **High-to-Low (Expanded)** |  |  |  |  |  |  |  |  |  |  |
| Response Option 1 | 6 | 5 | 4 | 2 | 3 | 9 | 7 | 3 | 2 | 4 |
| Response Option 2 | 26 | 19 | 35 | 26 | 35 | 60 | 56 | 52 | 22 | 33 |
| Response Option 3 | 228 | 171 | 201 | 220 | 182 | 177 | 192 | 178 | 147 | 194 |
| Response Option 4 | 29 | 94 | 49 | 41 | 69 | 43 | 34 | 56 | 118 | 58 |
| **Half-Half (Expanded)** |  |  |  |  |  |  |  |  |  |  |
| Response Option 1 | 12 | 19 | 9 | 7 | 2 | 21 | 9 | 4 | 3 | 6 |
| Response Option 2 | 38 | 24 | 40 | 47 | 36 | 76 | 75 | 79 | 33 | 41 |
| Response Option 3 | 210 | 174 | 183 | 186 | 183 | 165 | 180 | 159 | 135 | 176 |
| Response Option 4 | 29 | 72 | 57 | 49 | 68 | 27 | 25 | 47 | 118 | 66 |

*Note*: The negatively worded items in the original Likert version have been reverse-coded so that a lower response option number indicates a lower endorsement on the construct. All items in the *High-to-Low* Expanded version and some items in the *Half-Half* Expanded have been reverse-coded so that a lower response option number always indicates a lower endorsement on the construct. This means the order of the response option in this table is not the same as the one presented to the participant. For example, for the *High-to-Low* version, “Response Option 4” is presented as the first option to the participant whereas “Response Option 1” is presented as the last option.

**Table B: Distributions of the Response Options for the Conscientiousness Scale**

|  | **Item 1** | **Item 2** | **Item 3** | **Item 4** | **Item 5** | **Item 6** | **Item 7** | **Item 8** | **Item 9** |
| --- | --- | --- | --- | --- | --- | --- | --- | --- | --- |
| **Original (Likert)** |  |  |  |  |  |  |  |  |  |
| Response Option 1 | 0 | 17 | 1 | 9 | 23 | 5 | 5 | 6 | 47 |
| Response Option 2 | 23 | 130 | 14 | 102 | 128 | 32 | 56 | 57 | 135 |
| Response Option 3 | 126 | 97 | 99 | 112 | 98 | 143 | 139 | 128 | 85 |
| Response Option 4 | 141 | 46 | 176 | 67 | 41 | 110 | 90 | 99 | 23 |
| **Low-to-High (Expanded)** |  |  |  |  |  |  |  |  |  |
| Response Option 1 | 4 | 9 | 3 | 9 | 18 | 6 | 8 | 6 | 27 |
| Response Option 2 | 24 | 66 | 8 | 38 | 80 | 26 | 40 | 59 | 124 |
| Response Option 3 | 134 | 115 | 95 | 137 | 126 | 149 | 168 | 166 | 119 |
| Response Option 4 | 128 | 100 | 184 | 106 | 66 | 109 | 74 | 59 | 20 |
| **High-to-Low (Expanded)** |  |  |  |  |  |  |  |  |  |
| Response Option 1 | 1 | 3 | 0 | 4 | 10 | 1 | 6 | 5 | 26 |
| Response Option 2 | 16 | 36 | 6 | 39 | 52 | 24 | 41 | 47 | 125 |
| Response Option 3 | 140 | 110 | 97 | 149 | 124 | 158 | 157 | 174 | 115 |
| Response Option 4 | 133 | 141 | 187 | 98 | 104 | 107 | 86 | 64 | 24 |
| **Half-Half (Expanded)** |  |  |  |  |  |  |  |  |  |
| Response Option 1 | 0 | 4 | 5 | 3 | 11 | 8 | 4 | 10 | 28 |
| Response Option 2 | 23 | 46 | 10 | 61 | 55 | 30 | 54 | 52 | 130 |
| Response Option 3 | 136 | 110 | 100 | 131 | 134 | 150 | 170 | 180 | 115 |
| Response Option 4 | 130 | 129 | 174 | 94 | 89 | 101 | 61 | 47 | 16 |

*Note*: The negatively worded items in the original Likert version have been reverse-coded so that a lower response option number indicates a lower endorsement on the construct. All items in the *High-to-Low* Expanded version and some items in the *Half-Half* Expanded have been reverse-coded so that a lower response option number always indicates a lower endorsement on the construct. This means the order of the response option in this table is not the same as the one presented to the participant. For example, for the *High-to-Low* version, “Response Option 4” is presented as the first option to the participant whereas “Response Option 1” is presented as the last option.

**Table C: Distributions of the Response Options for the Extraversion Scale**

|  | **Item 1** | **Item 2** | **Item 3** | **Item 4** | **Item 5** | **Item 6** | **Item 7** | **Item 8** |
| --- | --- | --- | --- | --- | --- | --- | --- | --- |
| **Original (Likert)** |  |  |  |  |  |  |  |  |
| Response Option 1 | 26 | 51 | 10 | 14 | 54 | 40 | 46 | 20 |
| Response Option 2 | 84 | 151 | 81 | 90 | 132 | 109 | 163 | 72 |
| Response Option 3 | 127 | 75 | 153 | 134 | 87 | 102 | 65 | 153 |
| Response Option 4 | 56 | 16 | 49 | 55 | 20 | 42 | 19 | 48 |
| **Low-to-High (Expanded)** |  |  |  |  |  |  |  |  |
| Response Option 1 | 10 | 27 | 11 | 6 | 34 | 12 | 11 | 8 |
| Response Option 2 | 91 | 125 | 123 | 66 | 147 | 110 | 75 | 94 |
| Response Option 3 | 141 | 91 | 141 | 172 | 91 | 133 | 158 | 139 |
| Response Option 4 | 49 | 48 | 16 | 47 | 19 | 36 | 47 | 50 |
| **High-to-Low (Expanded)** |  |  |  |  |  |  |  |  |
| Response Option 1 | 13 | 24 | 12 | 6 | 35 | 16 | 14 | 6 |
| Response Option 2 | 74 | 94 | 89 | 68 | 132 | 112 | 70 | 67 |
| Response Option 3 | 142 | 108 | 160 | 165 | 93 | 132 | 164 | 150 |
| Response Option 4 | 60 | 63 | 28 | 50 | 29 | 29 | 41 | 66 |
| **Half-Half (Expanded)** |  |  |  |  |  |  |  |  |
| Response Option 1 | 12 | 22 | 11 | 10 | 30 | 19 | 10 | 8 |
| Response Option 2 | 84 | 98 | 105 | 61 | 128 | 111 | 68 | 97 |
| Response Option 3 | 130 | 106 | 145 | 151 | 96 | 123 | 172 | 131 |
| Response Option 4 | 60 | 60 | 25 | 64 | 32 | 33 | 36 | 50 |

*Note*: The negatively worded items in the original Likert version have been reverse-coded so that a lower response option number indicates a lower endorsement on the construct. All items in the *High-to-Low* Expanded version and some items in the *Half-Half* Expanded have been reverse-coded so that a lower response option number always indicates a lower endorsement on the construct. This means the order of the response option in this table is not the same as the one presented to the participant. For example, for the *High-to-Low* version, “Response Option 4” is presented as the first option to the participant whereas “Response Option 1” is presented as the last option.

**Table D: Distributions of the Response Options for the Neuroticism Scale**

|  | **Item 1** | **Item 2** | **Item 3** | **Item 4** | **Item 5** | **Item 6** | **Item 7** | **Item 8** |
| --- | --- | --- | --- | --- | --- | --- | --- | --- |
| **Original (Likert)** |  |  |  |  |  |  |  |  |
| Response Option 1 | 114 | 39 | 20 | 14 | 42 | 25 | 40 | 29 |
| Response Option 2 | 80 | 129 | 66 | 68 | 126 | 76 | 147 | 85 |
| Response Option 3 | 81 | 98 | 115 | 145 | 101 | 154 | 88 | 113 |
| Response Option 4 | 17 | 26 | 91 | 65 | 23 | 37 | 17 | 65 |
| **Low-to-High (Expanded)** |  |  |  |  |  |  |  |  |
| Response Option 1 | 65 | 12 | 14 | 33 | 37 | 58 | 45 | 12 |
| Response Option 2 | 171 | 171 | 110 | 164 | 142 | 174 | 153 | 94 |
| Response Option 3 | 49 | 95 | 112 | 84 | 100 | 49 | 78 | 147 |
| Response Option 4 | 6 | 13 | 55 | 10 | 12 | 10 | 15 | 38 |
| **High-to-Low (Expanded)** |  |  |  |  |  |  |  |  |
| Response Option 1 | 58 | 17 | 14 | 30 | 40 | 62 | 29 | 9 |
| Response Option 2 | 150 | 157 | 110 | 156 | 141 | 164 | 131 | 88 |
| Response Option 3 | 70 | 90 | 116 | 82 | 87 | 46 | 93 | 140 |
| Response Option 4 | 10 | 24 | 48 | 20 | 20 | 16 | 35 | 51 |
| **Half-Half (Expanded)** |  |  |  |  |  |  |  |  |
| Response Option 1 | 82 | 11 | 6 | 28 | 31 | 61 | 22 | 5 |
| Response Option 2 | 144 | 146 | 104 | 152 | 131 | 167 | 113 | 87 |
| Response Option 3 | 52 | 105 | 119 | 93 | 105 | 50 | 119 | 153 |
| Response Option 4 | 10 | 26 | 59 | 15 | 21 | 10 | 34 | 43 |

*Note*: The negatively worded items in the original Likert version have been reverse-coded so that a lower response option number indicates a lower endorsement on the construct. All items in the *High-to-Low* Expanded version and some items in the *Half-Half* Expanded have been reverse-coded so that a lower response option number always indicates a lower endorsement on the construct. This means the order of the response option in this table is not the same as the one presented to the participant. For example, for the *High-to-Low* version, “Response Option 4” is presented as the first option to the participant whereas “Response Option 1” is presented as the last option.

**Table F: Distributions of the Response Options for the Openness Scale**

|  | **Item 1** | **Item 2** | **Item 3** | **Item 4** | **Item 5** | **Item 6** | **Item 7** | **Item 8** | **Item 9** | **Item 10** |
| --- | --- | --- | --- | --- | --- | --- | --- | --- | --- | --- |
| **Original (Likert)** |  |  |  |  |  |  |  |  |  |  |
| Response Option 1 | 6 | 11 | 15 | 7 | 63 | 13 | 4 | 20 | 38 | 4 |
| Response Option 2 | 68 | 55 | 119 | 50 | 129 | 80 | 55 | 94 | 99 | 23 |
| Response Option 3 | 170 | 126 | 133 | 128 | 87 | 138 | 146 | 128 | 116 | 101 |
| Response Option 4 | 47 | 99 | 24 | 106 | 12 | 60 | 86 | 49 | 38 | 163 |
| **Low-to-High (Expanded)** |  |  |  |  |  |  |  |  |  |  |
| Response Option 1 | 11 | 7 | 10 | 2 | 40 | 3 | 4 | 26 | 21 | 3 |
| Response Option 2 | 47 | 38 | 90 | 41 | 144 | 35 | 41 | 70 | 87 | 32 |
| Response Option 3 | 175 | 145 | 154 | 131 | 97 | 199 | 169 | 114 | 155 | 109 |
| Response Option 4 | 57 | 100 | 36 | 116 | 9 | 53 | 76 | 80 | 27 | 146 |
| **High-to-Low (Expanded)** |  |  |  |  |  |  |  |  |  |  |
| Response Option 1 | 2 | 7 | 16 | 6 | 37 | 5 | 4 | 19 | 29 | 1 |
| Response Option 2 | 50 | 40 | 90 | 51 | 144 | 40 | 34 | 69 | 83 | 33 |
| Response Option 3 | 182 | 159 | 160 | 123 | 93 | 183 | 157 | 136 | 148 | 97 |
| Response Option 4 | 55 | 83 | 23 | 109 | 15 | 61 | 94 | 65 | 29 | 158 |
| **Half-Half (Expanded)** |  |  |  |  |  |  |  |  |  |  |
| Response Option 1 | 8 | 4 | 18 | 4 | 31 | 5 | 4 | 27 | 15 | 1 |
| Response Option 2 | 53 | 44 | 80 | 40 | 162 | 40 | 43 | 84 | 86 | 40 |
| Response Option 3 | 180 | 151 | 152 | 122 | 83 | 191 | 164 | 118 | 148 | 116 |
| Response Option 4 | 48 | 90 | 39 | 123 | 13 | 53 | 78 | 60 | 40 | 132 |

*Note*: The negatively worded items in the original Likert version have been reverse-coded so that a lower response option number indicates a lower endorsement on the construct. All items in the *High-to-Low* Expanded version and some items in the *Half-Half* Expanded have been reverse-coded so that a lower response option number always indicates a lower endorsement on the construct. This means the order of the response option in this table is not the same as the one presented to the participant. For example, for the *High-to-Low* version, “Response Option 4” is presented as the first option to the participant whereas “Response Option 1” is presented as the last option.

**Table G: Distributions of the Response Options for the Agreeableness Scale**

|  | **Item 1** | **Item 2** | **Item 3** | **Item 4** | **Item 5** | **Item 6** | **Item 7** | **Item 8** | **Item 9** |
| --- | --- | --- | --- | --- | --- | --- | --- | --- | --- |
| **Original (Likert)** |  |  |  |  |  |  |  |  |  |
| Response Option 1 | 21 | 2 | 2 | 7 | 10 | 22 | 7 | 7 | 4 |
| Response Option 2 | 122 | 41 | 41 | 64 | 41 | 88 | 24 | 86 | 28 |
| Response Option 3 | 108 | 167 | 91 | 134 | 130 | 107 | 143 | 114 | 133 |
| Response Option 4 | 35 | 76 | 152 | 81 | 105 | 69 | 112 | 79 | 121 |
| **Low-to-High (Expanded)** |  |  |  |  |  |  |  |  |  |
| Response Option 1 | 35 | 1 | 3 | 13 | 10 | 18 | 1 | 1 | 4 |
| Response Option 2 | 176 | 15 | 37 | 40 | 104 | 109 | 12 | 33 | 33 |
| Response Option 3 | 68 | 162 | 109 | 136 | 136 | 115 | 121 | 126 | 189 |
| Response Option 4 | 14 | 115 | 144 | 104 | 43 | 51 | 159 | 133 | 67 |
| **High-to-Low (Expanded)** |  |  |  |  |  |  |  |  |  |
| Response Option 1 | 33 | 0 | 2 | 7 | 6 | 12 | 0 | 0 | 2 |
| Response Option 2 | 173 | 12 | 51 | 48 | 73 | 117 | 2 | 27 | 28 |
| Response Option 3 | 77 | 192 | 106 | 145 | 152 | 107 | 153 | 127 | 189 |
| Response Option 4 | 16 | 95 | 140 | 99 | 68 | 63 | 144 | 145 | 80 |
| **Half-Half (Expanded)** |  |  |  |  |  |  |  |  |  |
| Response Option 1 | 49 | 3 | 3 | 9 | 13 | 15 | 3 | 1 | 7 |
| Response Option 2 | 155 | 22 | 42 | 63 | 108 | 131 | 5 | 36 | 31 |
| Response Option 3 | 63 | 152 | 112 | 109 | 127 | 86 | 130 | 123 | 174 |
| Response Option 4 | 14 | 104 | 124 | 100 | 33 | 49 | 143 | 121 | 69 |

*Note*: The negatively worded items in the original Likert version have been reverse-coded so that a lower response option number indicates a lower endorsement on the construct. All items in the *High-to-Low* Expanded version and some items in the *Half-Half* Expanded have been reverse-coded so that a lower response option number always indicates a lower endorsement on the construct. This means the order of the response option in this table is not the same as the one presented to the participant. For example, for the *High-to-Low* version, “Response Option 4” is presented as the first option to the participant whereas “Response Option 1” is presented as the last option.
