# Supplementary material for: Improved Properties of the Big Five Inventory and the Rosenberg Self-Esteem Scale in the Expanded Format Relative to the Likert Format
Source: Front Psychol. 2019 Jun 4;10:1286. doi: 10.3389/fpsyg.2019.01286 (PMC6558198; doi:10.3389/fpsyg.2019.01286)
Supplement: Supplementary file 4 [file Table_4.DOCX]

**Factor Loadings and Factor Correlations from the Confirmatory Factor Analyses for All Versions of the Big Five Inventory and the Rosenberg Self-Esteem Scale**

**Table A: Standardized factor loadings of the 1-Factor Model for the Rosenberg Self-Esteem Scale**

|  | **Original (Likert)** | **Low-to-High (Expanded)** | **High-to-Low (Expanded)** | **Half-Half (Expanded)** |
| --- | --- | --- | --- | --- |
| **Item 1** | 0.83 | 0.77 | 0.72 | 0.70 |
| **Item 2** | 0.88 | 0.78 | 0.76 | 0.69 |
| **Item 3** | 0.82 | 0.89 | 0.82 | 0.82 |
| **Item 4** | 0.65 | 0.62 | 0.65 | 0.54 |
| **Item 5** | 0.73 | 0.82 | 0.86 | 0.83 |
| **Item 6** | 0.86 | 0.86 | 0.84 | 0.86 |
| **Item 7** | 0.82 | 0.94 | 0.87 | 0.91 |
| **Item 8** | 0.70 | 0.87 | 0.74 | 0.73 |
| **Item 9** | 0.67 | 0.80 | 0.79 | 0.77 |
| **Item 10** | 0.81 | 0.85 | 0.83 | 0.83 |

Note: For the CFA models, the diagonally weighted least squares estimator with robust corrections was used because all items were measured on a 4-point scale and thus are treated as ordinal data.

**Table B: Standardized factor loadings of the 2-Factor Model for the Rosenberg Self-Esteem Scale**

|  | **Original (Likert)** | | **Low-to-High (Expanded)** | | **High-to-Low (Expanded)** | | **Half-Half (Expanded)** | |
| --- | --- | --- | --- | --- | --- | --- | --- | --- |
|  | PW | NW | PW | NW | PW | NW | PW | NW |
| **Item 1** | 0.85 |  | 0.77 |  | 0.72 |  | 0.71 |  |
| **Item 2** | 0.90 |  | 0.78 |  | 0.76 |  | 0.70 |  |
| **Item 4** | 0.67 |  | 0.62 |  | 0.65 |  | 0.55 |  |
| **Item 6** | 0.89 |  | 0.86 |  | 0.84 |  | 0.87 |  |
| **Item 7** | 0.84 |  | 0.94 |  | 0.87 |  | 0.93 |  |
| **Item 3** |  | 0.88 |  | 0.89 |  | 0.82 |  | 0.84 |
| **Item 5** |  | 0.78 |  | 0.82 |  | 0.86 |  | 0.84 |
| **Item 8** |  | 0.74 |  | 0.87 |  | 0.74 |  | 0.74 |
| **Item 9** |  | 0.70 |  | 0.80 |  | 0.79 |  | 0.79 |
| **Item 10** |  | 0.85 |  | 0.85 |  | 0.83 |  | 0.85 |
| **Factor Correlation** | 0.80 | | 1.00 | | 1.00 | | 0.93 | |

Note: PW = Positively Word Factor; NW = Negative Word Factor. For the CFA models, the diagonally weighted least squares estimator with robust corrections was used because all items were measured on a 4-point scale and thus are treated as ordinal data.

**Table C: Standardized factor loadings of the 1-Factor Model for the Conscientiousness Scale**

|  | **Original (Likert)** | **Low-to-High (Expanded)** | **High-to-Low (Expanded)** | **Half-Half (Expanded)** |
| --- | --- | --- | --- | --- |
| **Item 1** | 0.69 | 0.83 | 0.83 | 0.77 |
| **Item 2** | 0.53 | 0.67 | 0.53 | 0.54 |
| **Item 3** | 0.73 | 0.74 | 0.78 | 0.70 |
| **Item 4** | 0.62 | 0.64 | 0.59 | 0.55 |
| **Item 5** | 0.54 | 0.78 | 0.77 | 0.68 |
| **Item 6** | 0.59 | 0.66 | 0.61 | 0.50 |
| **Item 7** | 0.65 | 0.66 | 0.69 | 0.55 |
| **Item 8** | 0.71 | 0.70 | 0.61 | 0.56 |
| **Item 9** | 0.53 | 0.69 | 0.65 | 0.49 |

Note: For the CFA models, the diagonally weighted least squares estimator with robust corrections was used because all items were measured on a 4-point scale and thus are treated as ordinal data.

**Table D: Standardized factor loadings of the 2-Factor Model for the Conscientiousness Scale**

|  | **Original (Likert)** | | **Low-to-High (Expanded)** | | **High-to-Low (Expanded)** | | **Half-Half (Expanded)** | |
| --- | --- | --- | --- | --- | --- | --- | --- | --- |
|  | PW | NW | PW | NW | PW | NW | PW | NW |
| **Item 1** | 0.71 |  | 0.83 |  | 0.84 |  | 0.80 |  |
| **Item 3** | 0.75 |  | 0.75 |  | 0.79 |  | 0.73 |  |
| **Item 6** | 0.61 |  | 0.67 |  | 0.62 |  | 0.52 |  |
| **Item 7** | 0.67 |  | 0.66 |  | 0.70 |  | 0.57 |  |
| **Item 8** | 0.74 |  | 0.70 |  | 0.62 |  | 0.59 |  |
| **Item 2** |  | 0.59 |  | 0.68 |  | 0.54 |  | 0.58 |
| **Item 4** |  | 0.71 |  | 0.65 |  | 0.60 |  | 0.59 |
| **Item 5** |  | 0.60 |  | 0.79 |  | 0.79 |  | 0.75 |
| **Item 9** |  | 0.59 |  | 0.70 |  | 0.66 |  | 0.51 |
| **Factor Correlation** | 0.75 | | 0.96 | | 0.94 | | 0.80 | |

Note: PW = Positively Word Factor; NW = Negative Word Factor. For the CFA models, the diagonally weighted least squares estimator with robust corrections was used because all items were measured on a 4-point scale and thus are treated as ordinal data.

**Table E: Standardized factor loadings of the 1-Factor Model for the Extraversion Scale**

|  | **Original (Likert)** | **Low-to-High (Expanded)** | **High-to-Low (Expanded)** | **Half-Half (Expanded)** |
| --- | --- | --- | --- | --- |
| **Item 1** | 0.78 | 0.87 | 0.79 | 0.87 |
| **Item 2** | 0.80 | 0.72 | 0.66 | 0.54 |
| **Item 3** | 0.74 | 0.74 | 0.72 | 0.74 |
| **Item 4** | 0.76 | 0.73 | 0.74 | 0.79 |
| **Item 5** | 0.85 | 0.85 | 0.79 | 0.70 |
| **Item 6** | 0.42 | 0.56 | 0.46 | 0.50 |
| **Item 7** | 0.63 | 0.79 | 0.78 | 0.71 |
| **Item 8** | 0.78 | 0.79 | 0.84 | 0.81 |

Note: For the CFA models, the diagonally weighted least squares estimator with robust corrections was used because all items were measured on a 4-point scale and thus are treated as ordinal data.

**Table F: Standardized factor loadings of the 2-Factor Model for the Extraversion Scale**

|  | **Original (Likert)** | | **Low-to-High (Expanded)** | | **High-to-Low (Expanded)** | | **Half-Half (Expanded)** | |
| --- | --- | --- | --- | --- | --- | --- | --- | --- |
|  | PW | NW | PW | NW | PW | NW | PW | NW |
| **Item 1** | 0.84 |  | 0.87 |  | 0.79 |  | 0.88 |  |
| **Item 3** | 0.77 |  | 0.74 |  | 0.73 |  | 0.75 |  |
| **Item 4** | 0.80 |  | 0.73 |  | 0.75 |  | 0.80 |  |
| **Item 6** | 0.44 |  | 0.56 |  | 0.46 |  | 0.50 |  |
| **Item 8** | 0.81 |  | 0.79 |  | 0.84 |  | 0.81 |  |
| **Item 2** |  | 0.85 |  | 0.72 |  | 0.66 |  | 0.57 |
| **Item 5** |  | 0.94 |  | 0.86 |  | 0.80 |  | 0.74 |
| **Item 7** |  | 0.67 |  | 0.79 |  | 0.79 |  | 0.74 |
| **Factor Correlation** | 0.74 | | 1.00 | | 0.97 | | 0.91 | |

Note: PW = Positively Word Factor; NW = Negative Word Factor. For the CFA models, the diagonally weighted least squares estimator with robust corrections was used because all items were measured on a 4-point scale and thus are treated as ordinal data.

**Table G: Standardized factor loadings of the 1-Factor Model for the Neuroticism Scale**

|  | **Original (Likert)** | **Low-to-High (Expanded)** | **High-to-Low (Expanded)** | **Half-Half (Expanded)** |
| --- | --- | --- | --- | --- |
| **Item 1** | 0.57 | 0.55 | 0.66 | 0.60 |
| **Item 2** | 0.78 | 0.74 | 0.82 | 0.80 |
| **Item 3** | 0.85 | 0.75 | 0.79 | 0.75 |
| **Item 4** | 0.80 | 0.83 | 0.84 | 0.69 |
| **Item 5** | 0.67 | 0.72 | 0.74 | 0.75 |
| **Item 6** | 0.57 | 0.63 | 0.69 | 0.65 |
| **Item 7** | 0.54 | 0.51 | 0.71 | 0.57 |
| **Item 8** | 0.72 | 0.71 | 0.75 | 0.64 |

Note: For the CFA models, the diagonally weighted least squares estimator with robust corrections was used because all items were measured on a 4-point scale and thus are treated as ordinal data.

**Table H: Standardized factor loadings of the 2-Factor Model for the Neuroticism Scale**

|  | **Original (Likert)** | | **Low-to-High (Expanded)** | | **High-to-Low (Expanded)** | | **Half-Half (Expanded)** | |
| --- | --- | --- | --- | --- | --- | --- | --- | --- |
|  | PW | NW | PW | NW | PW | NW | PW | NW |
| **Item 1** | 0.59 |  | 0.55 |  | 0.66 |  | 0.61 |  |
| **Item 3** | 0.87 |  | 0.76 |  | 0.80 |  | 0.76 |  |
| **Item 4** | 0.82 |  | 0.84 |  | 0.84 |  | 0.70 |  |
| **Item 6** | 0.59 |  | 0.64 |  | 0.69 |  | 0.66 |  |
| **Item 8** | 0.73 |  | 0.71 |  | 0.75 |  | 0.65 |  |
| **Item 2** |  | 0.87 |  | 0.77 |  | 0.83 |  | 0.82 |
| **Item 5** |  | 0.73 |  | 0.75 |  | 0.75 |  | 0.77 |
| **Item 7** |  | 0.58 |  | 0.53 |  | 0.72 |  | 0.59 |
| **Factor Correlation** | 0.80 | | 0.93 | | 0.97 | | 0.94 | |

Note: PW = Positively Word Factor; NW = Negative Word Factor. For the CFA models, the diagonally weighted least squares estimator with robust corrections was used because all items were measured on a 4-point scale and thus are treated as ordinal data.

**Table I: Standardized factor loadings of the 1-Factor Model for the Openness Scale**

|  | **Original (Likert)** | **Low-to-High (Expanded)** | **High-to-Low (Expanded)** | **Half-Half (Expanded)** |
| --- | --- | --- | --- | --- |
| **Item 1** | 0.80 | 0.78 | 0.81 | 0.84 |
| **Item 2** | 0.84 | 0.78 | 0.68 | 0.82 |
| **Item 3** | 0.74 | 0.68 | 0.59 | 0.71 |
| **Item 4** | 0.67 | 0.61 | 0.65 | 0.54 |
| **Item 5** | 0.12 | 0.33 | 0.24 | 0.14 |
| **Item 6** | 0.56 | 0.62 | 0.65 | 0.70 |
| **Item 7** | 0.63 | 0.65 | 0.61 | 0.60 |
| **Item 8** | 0.40 | 0.81 | 0.80 | 0.54 |
| **Item 9** | 0.50 | 0.65 | 0.75 | 0.53 |
| **Item 10** | 0.62 | 0.50 | 0.58 | 0.53 |

Note: For the CFA models, the diagonally weighted least squares estimator with robust corrections was used because all items were measured on a 4-point scale and thus are treated as ordinal data.

**Table J: Standardized factor loadings of the 1-Factor Model for the Agreeableness Scale**

|  | **Original (Likert)** | **Low-to-High (Expanded)** | **High-to-Low (Expanded)** | **Half-Half (Expanded)** |
| --- | --- | --- | --- | --- |
| **Item 1** | 0.40 | 0.49 | 0.35 | 0.27 |
| **Item 2** | 0.61 | 0.60 | 0.49 | 0.72 |
| **Item 3** | 0.56 | 0.50 | 0.41 | 0.35 |
| **Item 4** | 0.59 | 0.67 | 0.56 | 0.58 |
| **Item 5** | 0.49 | 0.52 | 0.57 | 0.42 |
| **Item 6** | 0.50 | 0.64 | 0.44 | 0.46 |
| **Item 7** | 0.78 | 0.75 | 0.68 | 0.87 |
| **Item 8** | 0.55 | 0.60 | 0.43 | 0.40 |
| **Item 9** | 0.62 | 0.64 | 0.47 | 0.66 |

Note: For the CFA models, the diagonally weighted least squares estimator with robust corrections was used because all items were measured on a 4-point scale and thus are treated as ordinal data.

**Table K: Standardized factor loadings of the 2-Factor Model for the Agreeableness Scale**

|  | **Original (Likert)** | | **Low-to-High (Expanded)** | | **High-to-Low (Expanded)** | | **Half-Half (Expanded)** | |
| --- | --- | --- | --- | --- | --- | --- | --- | --- |
|  | PW | NW | PW | NW | PW | NW | PW | NW |
| **Item 2** | 0.63 |  | 0.61 |  | 0.52 |  | 0.73 |  |
| **Item 4** | 0.61 |  | 0.68 |  | 0.60 |  | 0.59 |  |
| **Item 5** | 0.50 |  | 0.53 |  | 0.59 |  | 0.43 |  |
| **Item 7** | 0.81 |  | 0.76 |  | 0.72 |  | 0.88 |  |
| **Item 9** | 0.63 |  | 0.65 |  | 0.49 |  | 0.68 |  |
| **Item 1** |  | 0.46 |  | 0.51 |  | 0.41 |  | 0.38 |
| **Item 3** |  | 0.64 |  | 0.52 |  | 0.54 |  | 0.52 |
| **Item 6** |  | 0.56 |  | 0.66 |  | 0.55 |  | 0.57 |
| **Item 8** |  | 0.63 |  | 0.63 |  | 0.56 |  | 0.58 |
| **Factor Correlation** | 0.74 | | 0.90 | | 0.60 | | 0.56 | |

Note: PW = Positively Word Factor; NW = Negative Word Factor. For the CFA models, the diagonally weighted least squares estimator with robust corrections was used because all items were measured on a 4-point scale and thus are treated as ordinal data.
