# Supplementary material for: Improved Properties of the Big Five Inventory and the Rosenberg Self-Esteem Scale in the Expanded Format Relative to the Likert Format
Source: Front Psychol. 2019 Jun 4;10:1286. doi: 10.3389/fpsyg.2019.01286 (PMC6558198; doi:10.3389/fpsyg.2019.01286)
Supplement: Supplementary file 5 [file Table_5.DOCX]

**Parallel Analyses for All Scales**

**Figure A: Parallel Analysis for All Versions of the Rosenberg Self-Esteem Scale**

**Figure B: Parallel Analysis for All Versions of the Conscientiousness Scale**

**Figure C: Parallel Analysis for All Versions of the Neuroticism Scale**

**Figure D: Parallel Analysis for All Versions of the Extraversion Scale**

**Figure E: Parallel Analysis for All Versions of the Openness Scale**

**Figure F: Parallel Analysis for All Versions of the Agreeableness Scale**
