# Supplementary material for: Improved Properties of the Big Five Inventory and the Rosenberg Self-Esteem Scale in the Expanded Format Relative to the Likert Format
Source: Front Psychol. 2019 Jun 4;10:1286. doi: 10.3389/fpsyg.2019.01286 (PMC6558198; doi:10.3389/fpsyg.2019.01286)
Supplement: Supplementary file 6 [file Table_6.DOCX]

**Factor Loadings and Factor Correlations from the Exploratory Factor Analyses for the Neuroticism, Openness and Agreeableness Scales**

**Table A: Factor Loadings from the Exploratory Factor Analysis for the Neuroticism Scale**

|  | **Original** | | **Low-to-High** | | **High-to-Low** | | **Half-Half** | |
| --- | --- | --- | --- | --- | --- | --- | --- | --- |
| **Factor Loadings** |  |  |  |  |  |  |  |  |
|  | Factor 1 | Factor 2 | Factor 1 | Factor 2 | Factor 1 | Factor 2 | Factor 1 | Factor 2 |
| Item 1 | 0.63 | -0.11 | 0.09 | 0.52 | 0.02 | 0.71 | 0.18 | 0.47 |
| Item 2 | 0.59 | 0.29 | 0.43 | 0.37 | 0.56 | 0.31 | 0.48 | 0.39 |
| Item 3 | 0.88 | -0.04 | 0.93 | -0.11 | 0.94 | -0.14 | 0.84 | -0.04 |
| Item 4 | 0.86 | -0.08 | 0.65 | 0.22 | 0.71 | 0.15 | 0.79 | -0.03 |
| Item 5 | 0.51 | 0.24 | 0.04 | 0.79 | 0.09 | 0.70 | -0.04 | 0.91 |
| Item 6 | 0.60 | -0.06 | -0.05 | 0.77 | -0.01 | 0.77 | 0.01 | 0.67 |
| Item 7 | 0.00 | 1.00 | 0.31 | 0.22 | 0.54 | 0.18 | 0.31 | 0.31 |
| Item 8 | 0.62 | 0.21 | 0.61 | 0.15 | 0.57 | 0.19 | 0.47 | 0.21 |
| **Factor Correlation** | 0.41 | | 0.61 | | 0.72 | | 0.59 | |

*Note:* For the exploratory factor analysis, we used the polychoric correlation input matrix, least squares (labeled “minres” in the *psych* package) extraction method, and oblimin rotation. For each item, the highest loading has the darkest loading. High cross-loadings are shaded with a lighter color.

**Table B: Factor Loadings from the Exploratory Factor Analysis for the Openness Scale**

|  | **Original** | | | **Low-to-High** | | | **High-to-low** | | | **Half-Half** | | |
| --- | --- | --- | --- | --- | --- | --- | --- | --- | --- | --- | --- | --- |
| **Factor Loadings** |  |  |  |  |  |  |  |  |  |  |  |  |
|  | Factor 1 | Factor 2 | Factor 3 | Factor 1 | Factor 2 | Factor 3 | Factor 1 | Factor 2 | Factor 3 | Factor 1 | Factor 2 | Factor 3 |
| Item 1 | 0.91 | -0.06 | -0.04 | 0.79 | -0.11 | 0.16 | 0.65 | 0.06 | 0.21 | 0.94 | 0.07 | -0.07 |
| Item 2 | 0.65 | 0.18 | 0.12 | 0.76 | 0.16 | -0.03 | 0.84 | 0.04 | -0.07 | 0.61 | 0.08 | 0.22 |
| Item 3 | 0.75 | 0.01 | 0.01 | 0.76 | 0.03 | -0.04 | 0.52 | 0.04 | 0.12 | 0.63 | -0.14 | 0.27 |
| Item 4 | 0.02 | 0.85 | 0.04 | -0.15 | 0.66 | 0.21 | -0.06 | 0.69 | 0.14 | 0.06 | 0.72 | -0.02 |
| Item 5 | 0.12 | 0.16 | -0.17 | 0.28 | 0.05 | 0.00 | 0.11 | 0.11 | 0.07 | 0.02 | 0.01 | 0.12 |
| Item 6 | 0.05 | -0.08 | 0.74 | 0.25 | 0.10 | 0.40 | 0.33 | -0.11 | 0.56 | 0.12 | 0.03 | 0.72 |
| Item 7 | -0.04 | 0.09 | 0.75 | 0.07 | 0.03 | 0.74 | 0.02 | 0.05 | 0.73 | 0.13 | 0.15 | 0.46 |
| Item 8 | 0.01 | 0.61 | -0.15 | 0.08 | 0.90 | 0.02 | 0.09 | 0.94 | -0.08 | 0.11 | 0.68 | -0.04 |
| Item 9 | 0.03 | 0.48 | 0.11 | 0.02 | 0.77 | -0.08 | -0.04 | 0.76 | 0.09 | -0.13 | 0.59 | 0.27 |
| Item 10 | 0.25 | 0.13 | 0.36 | 0.00 | 0.03 | 0.63 | -0.02 | 0.16 | 0.59 | -0.04 | 0.06 | 0.63 |
| **Factor Correlations** |  |  |  |  |  |  |  |  |  |  |  |  |
|  | Factor 1 | Factor 2 | Factor 3 | Factor 1 | Factor 2 | Factor 3 | Factor 1 | Factor 2 | Factor 3 | Factor 1 | Factor 2 | Factor 3 |
| Factor 1 | 1.00 | 0.53 | 0.54 | 1.00 | 0.41 | 0.59 | 1.00 | 0.46 | 0.59 | 1.00 | 0.41 | 0.56 |
| Factor 2 | 0.53 | 1.00 | 0.48 | 0.41 | 1.00 | 0.43 | 0.46 | 1.00 | 0.35 | 0.41 | 1.00 | 0.43 |
| Factor 3 | 0.54 | 0.48 | 1.00 | 0.59 | 0.43 | 1.00 | 0.59 | 0.35 | 1.00 | 0.56 | 0.43 | 1.00 |

*Note:* For the exploratory factor analysis, we used the polychoric correlation input matrix, least squares (labeled “minres” in the *psych* package) extraction method, and oblimin rotation. For each item, the highest loading has the darkest loading. High cross-loadings are shaded with a lighter color.

**Table C: Factor Loadings from the Exploratory Factor Analysis for the Agreeableness Scale When Two Factors are Extracted**

|  | **Original** | | **Low-to-High** | | **High-to-Low** | | **Half-Half** | |
| --- | --- | --- | --- | --- | --- | --- | --- | --- |
| **Factor Loadings** |  |  |  |  |  |  |  |  |
|  | Factor 1 | Factor 2 | Factor 1 | Factor 2 | Factor 1 | Factor 2 | Factor 1 | Factor 2 |
| Item 1 | -0.12 | 0.59 | 0.46 | -0.03 | 0.28 | 0.03 | 0.07 | 0.27 |
| Item 2 | 0.58 | 0.06 | 0.61 | -0.04 | 0.53 | -0.02 | 0.75 | -0.02 |
| Item 3 | 0.06 | 0.61 | 0.29 | 0.33 | 0.23 | 0.32 | 0.02 | 0.59 |
| Item 4 | 0.38 | 0.25 | 0.77 | -0.10 | 0.62 | -0.07 | 0.50 | 0.11 |
| Item 5 | 0.48 | 0.03 | 0.56 | -0.03 | 0.62 | -0.11 | 0.40 | -0.05 |
| Item 6 | 0.24 | 0.31 | 0.51 | 0.15 | 0.31 | 0.25 | 0.38 | 0.12 |
| Item 7 | 0.87 | -0.04 | 0.58 | 0.19 | 0.61 | 0.16 | 0.77 | 0.16 |
| Item 8 | 0.12 | 0.52 | 0.00 | 1.00 | -0.01 | 1.00 | 0.01 | 0.78 |
| Item 9 | 0.56 | 0.09 | 0.55 | 0.11 | 0.42 | 0.11 | 0.75 | -0.17 |
| **Factor Correlation** | 0.63 | | 0.48 | | 0.29 | | 0.33 | |

*Note:* For the exploratory factor analysis, we used the polychoric correlation input matrix, least squares (labeled “minres” in the *psych* package) extraction method, and oblimin rotation. For each item, the highest loading has the darkest loading. High cross-loadings are shaded with a lighter color.

**Table D: Factor Loadings from the Exploratory Factor Analysis for the Agreeableness Scale When Three Factors are Extracted**

|  | **Original** | | | **Low-to-High** | | | **High-to-low** | | | **Half-Half** | | |
| --- | --- | --- | --- | --- | --- | --- | --- | --- | --- | --- | --- | --- |
| **Factor Loadings** |  |  |  |  |  |  |  |  |  |  |  |  |
|  | Factor 1 | Factor 2 | Factor 3 | Factor 1 | Factor 2 | Factor 3 | Factor 1 | Factor 2 | Factor 3 | Factor 1 | Factor 2 | Factor 3 |
| Item 1 | -0.15 | 0.51 | 0.18 | 0.29 | -0.03 | 0.24 | -0.08 | 0.14 | 0.36 | -0.18 | 0.26 | 0.47 |
| Item 2 | 0.67 | 0.11 | -0.14 | 0.37 | -0.03 | 0.32 | 0.60 | -0.09 | 0.05 | 0.80 | -0.02 | -0.06 |
| Item 3 | 0.09 | 0.66 | -0.05 | 0.35 | 0.43 | -0.16 | -0.16 | 0.47 | 0.34 | 0.00 | 0.56 | 0.11 |
| Item 4 | 0.36 | 0.21 | 0.12 | 0.82 | -0.04 | 0.00 | 0.21 | -0.04 | 0.50 | 0.31 | 0.09 | 0.38 |
| Item 5 | 0.40 | -0.01 | 0.19 | 0.53 | 0.02 | 0.03 | 0.06 | -0.05 | 0.70 | 0.12 | -0.09 | 0.56 |
| Item 6 | 0.01 | 0.22 | 0.51 | 0.01 | 0.03 | 0.81 | 0.17 | 0.31 | 0.15 | 0.23 | 0.12 | 0.29 |
| Item 7 | 0.78 | 0.01 | 0.10 | 0.40 | 0.24 | 0.22 | 0.77 | 0.09 | 0.03 | 0.81 | 0.16 | 0.00 |
| Item 8 | 0.12 | 0.53 | 0.03 | -0.03 | 0.93 | 0.04 | 0.08 | 0.81 | -0.07 | 0.06 | 0.79 | -0.02 |
| Item 9 | 0.37 | -0.01 | 0.47 | 0.35 | 0.15 | 0.23 | 0.24 | 0.13 | 0.23 | 0.62 | -0.17 | 0.22 |
| **Factor Correlations** |  |  |  |  |  |  |  |  |  |  |  |  |
|  | Factor 1 | Factor 2 | Factor 3 | Factor 1 | Factor 2 | Factor 3 | Factor 1 | Factor 2 | Factor 3 | Factor 1 | Factor 2 | Factor 3 |
| Factor 1 | 1.00 | 0.56 | 0.45 | 1.00 | 0.41 | 0.51 | 1.00 | 0.34 | 0.45 | 1.00 | 0.28 | 0.42 |
| Factor 2 | 0.56 | 1.00 | 0.36 | 0.41 | 1.00 | 0.46 | 0.34 | 1.00 | 0.21 | 0.28 | 1.00 | 0.16 |
| Factor 3 | 0.45 | 0.36 | 1.00 | 0.51 | 0.46 | 1.00 | 0.45 | 0.21 | 1.00 | 0.42 | 0.16 | 1.00 |

*Note:* For the exploratory factor analysis, we used the polychoric correlation input matrix, least squares (labeled “minres” in the *psych* package) extraction method, and oblimin rotation. For each item, the highest loading has the darkest loading. High cross-loadings are shaded with a lighter color.
