# Supplementary material for: Improved Properties of the Big Five Inventory and the Rosenberg Self-Esteem Scale in the Expanded Format Relative to the Likert Format
Source: Front Psychol. 2019 Jun 4;10:1286. doi: 10.3389/fpsyg.2019.01286 (PMC6558198; doi:10.3389/fpsyg.2019.01286)
Supplement: Supplementary file 7 [file Table_7.DOCX]

**Factor Loadings and Factor Correlations of Alternative Confirmatory Factor Models for the Neuroticism, Openness and Agreeableness Scales**

**Table A: Factor loadings of Alternative Confirmatory Factor Model for the Neuroticism Scale**

|  | **Original (Likert)** | | **Low-to-High (Expanded)** | | **High-to-Low (Expanded)** | | **Half-Half (Expanded)** | |
| --- | --- | --- | --- | --- | --- | --- | --- | --- |
|  | **Anxiety** | **Depression** | **Anxiety** | **Depression** | **Anxiety** | **Depression** | **Anxiety** | **Depression** |
| **Item 1** | 0.79 |  | 0.76 |  | 0.84 |  | 0.82 |  |
| **Item 2** | 0.86 |  | 0.77 |  | 0.80 |  | 0.77 |  |
| **Item 3** | 0.80 |  | 0.86 |  | 0.85 |  | 0.72 |  |
| **Item 4** | 0.54 |  | 0.53 |  | 0.72 |  | 0.59 |  |
| **Item 5** | 0.72 |  | 0.73 |  | 0.76 |  | 0.66 |  |
| **Item 6** |  | 0.59 |  | 0.63 |  | 0.73 |  | 0.66 |
| **Item 7** |  | 0.70 |  | 0.86 |  | 0.81 |  | 0.85 |
| **Item 8** |  | 0.60 |  | 0.73 |  | 0.76 |  | 0.71 |
| **Factor Correlation** | 0.94 | | 0.74 | | 0.82 | | 0.77 | |

Note: For the CFA models, the diagonally weighted least squares estimator with robust corrections was used because all items were measured on a 4-point scale and thus are treated as ordinal data.

**Table B: Factor loadings of Alternative Confirmatory Factor Model for the Openness Scale**

|  | **Original (Likert)** | | | **Low-to-High (Expanded)** | | | **High-to-low (Expanded)** | | | **Half-Half (Expanded)** | | |
| --- | --- | --- | --- | --- | --- | --- | --- | --- | --- | --- | --- | --- |
| **Factor Loadings** |  |  |  |  |  |  |  |  |  |  |  |  |
|  | **Creative** | **Artistic** | **Thinker** | **Creative** | **Artistic** | **Thinker** | **Creative** | **Artistic** | **Thinker** | **Creative** | **Artistic** | **Thinker** |
| Item 1 | 0.83 |  |  | 0.85 |  |  | 0.91 |  |  | 0.88 |  |  |
| Item 2 | 0.89 |  |  | 0.84 |  |  | 0.76 |  |  | 0.87 |  |  |
| Item 3 | 0.78 |  |  | 0.74 |  |  | 0.66 |  |  | 0.76 |  |  |
| Item 4 | 0.13 |  |  | 0.37 |  |  | 0.25 |  |  | 0.14 |  |  |
| Item 5 |  | 0.92 |  |  | 0.70 |  |  | 0.72 |  |  | 0.73 |  |
| Item 6 |  | 0.49 |  |  | 1.01 |  |  | 0.94 |  |  | 0.71 |  |
| Item 7 |  | 0.63 |  |  | 0.73 |  |  | 0.81 |  |  | 0.70 |  |
| Item 8 |  |  | 0.66 |  |  | 0.74 |  |  | 0.77 |  |  | 0.83 |
| Item 9 |  |  | 0.75 |  |  | 0.77 |  |  | 0.73 |  |  | 0.67 |
| Item 10 |  |  | 0.73 |  |  | 0.60 |  |  | 0.64 |  |  | 0.60 |
| **Factor Correlations** |  |  |  |  |  |  |  |  |  |  |  |  |
|  | **Creative** | **Artistic** | **Thinker** | **Creative** | **Artistic** | **Thinker** | **Creative** | **Artistic** | **Thinker** | **Creative** | **Artistic** | **Thinker** |
| Creative | 1.00 |  |  | 1.00 |  |  | 1.00 |  |  | 1.00 |  |  |
| Artistic | 0.59 | 1.00 |  | 0.47 | 1.00 |  | 0.54 | 1.00 |  | 0.50 | 1.00 |  |
| Factor 3 | 0.68 | 0.56 | 1.00 | 0.76 | 0.55 | 1.00 | 0.79 | 0.47 | 1.00 | 0.74 | 0.61 | 1.00 |

Note: For the CFA models, the diagonally weighted least squares estimator with robust corrections was used because all items were measured on a 4-point scale and thus are treated as ordinal data.

**Table C: Factor loadings of Alternative Confirmatory Factor Model for the Agreeableness Scale**

|  | **Original (Likert)** | **Low-to-High (Expanded)** | **High-to-Low (Expanded)** | **Half-Half (Expanded)** |
| --- | --- | --- | --- | --- |
| **Item 1** | 0.40 | 0.49 | 0.35 | 0.26 |
| **Item 2** | 0.62 | 0.60 | 0.50 | 0.73 |
| **Item 3** | 0.51 | 0.44 | 0.35 | 0.25 |
| **Item 4** | 0.60 | 0.68 | 0.58 | 0.59 |
| **Item 5** | 0.49 | 0.53 | 0.58 | 0.43 |
| **Item 6** | 0.50 | 0.64 | 0.44 | 0.47 |
| **Item 7** | 0.79 | 0.76 | 0.70 | 0.87 |
| **Item 8** | 0.50 | 0.56 | 0.37 | 0.31 |
| **Item 9** | 0.63 | 0.64 | 0.47 | 0.67 |
| **Correlated Residuals between Items 3 and 8** | 0.26 | 0.33 | 0.30 | 0.44 |

Note: For the CFA models, the diagonally weighted least squares estimator with robust corrections was used because all items were measured on a 4-point scale and thus are treated as ordinal data.
