# Supplementary material for: Improved Properties of the Big Five Inventory and the Rosenberg Self-Esteem Scale in the Expanded Format Relative to the Likert Format
Source: Front Psychol. 2019 Jun 4;10:1286. doi: 10.3389/fpsyg.2019.01286 (PMC6558198; doi:10.3389/fpsyg.2019.01286)
Supplement: Supplementary file 8 [file Table_8.DOCX]

**Summary of the model with NW items loading on a method factor**

|  | **The model with NW items loading on a method factor** | | | |  |  |
| --- | --- | --- | --- | --- | --- | --- |
|  | **χ^2^** | **CFI** | **RMSEA** | **SRMR** | **Δχ^2^ relative to 1-factor model** | **Δχ^2^ relative to 2-factor model** |
| **RSE** | (df=30) |  |  |  | (df=5) | (df=4) |
| Original (Likert) | 229.79 | 0.96 | 0.15 | 0.06 | 150.41 (*p*=.00) | 9.65 (*p*=.05) |
| Low-to-High (Expanded) | 113.41 | 0.99 | 0.10 | 0.04 | 31.57 (*p*=.00) | 35.56 (*p*=.00) |
| High-to-Low (Expanded) | 136.54 | 0.98 | 0.11 | 0.06 | 44.01 (*p*=.00) | 41.05 (*p*=.00) |
| Half-Half (Expanded) | 162.52 | 0.97 | 0.12 | 0.06 | 26.44 (*p*=.00) | 5.02 (*p*=.28) |
| **Conscientiousness** | (df=23) |  |  |  | (df=4) | (df=3) |
| Original (Likert) | 100.98 | 0.93 | 0.11 | 0.07 | 33.16 (*p*=.00) | 0.87 (*p*=.83) |
| Low-to-High (Expanded) | N/A | N/A | N/A | N/A | N/A | N/A |
| High-to-Low (Expanded) | 73.23 | 0.97 | 0.09 | 0.06 | 9.66 (*p*=.05) | 6.77 (*p*=.08) |
| Half-Half (Expanded) | 33.32 | 0.98 | 0.04 | 0.04 | 20.36 (*p*=.00) | 0.46 (*p*=.93) |
| **Extraversion** | (df=17) |  |  |  | (df=3) | (df=2) |
| Original (Likert) | 205.32 | 0.93 | 0.20 | 0.09 | 79.37 (*p*=.00) | 0.63 (*p*=0.73) |
| Low-to-High (Expanded) | 93.88 | 0.98 | 0.13 | 0.06 | 0.53 (*p*=.91) | 0.50 (*p*=0.78) |
| High-to-Low (Expanded) | 104.21 | 0.96 | 0.13 | 0.05 | 1.85 (*p*=.60) | 0.56 (*p*=0.97) |
| Half-Half (Expanded) | N/A | N/A | N/A | N/A | N/A | N/A |
| **Neuroticism** | (df=17) |  |  |  | (df=3) | (df=2) |
| Original (Likert) | 82.53 | 0.96 | 0.12 | 0.06 | 50.28 (*p*=.00) | 5.14 (*p*=0.07) |
| Low-to-High (Expanded) | 138.03 | 0.93 | 0.16 | 0.08 | 4.26(*p*=.23) | 0.28 (*p*=0.87) |
| High-to-Low (Expanded) | 153.11 | 0.94 | 0.17 | 0.07 | 1.58 (*p*=.66) | 0.21 (*p*=0.90) |
| Half-Half (Expanded) | 137.56 | 0.92 | 0.16 | 0.07 | 6.42 (*p*=.02) | 0.26 (*p*=0.88) |
| **Agreeableness** | (df=23) |  |  |  | (df=4) | (df=3) |
| Original (Likert) | 39.10 | 0.98 | 0.05 | 0.05 | 26.70 (*p*=.00) | 4.77 (*p*=.19) |
| Low-to-High (Expanded) | N/A | N/A | N/A | N/A | N/A | N/A |
| High-to-Low (Expanded) | 48.74 | 0.94 | 0.06 | 0.06 | 27.4 (*p*=.00) | 5.43 (*p*=.14) |
| Half-Half (Expanded) | 64.37 | 0.95 | 0.08 | 0.06 | 68.8 (*p*=.00) | 25.01 (*p*=.00) |

Note: All models are estimated using *lavaan* (version 0.6-3). All χ^2^ tests are significant at *p*<0.01. Models with N/A are those with warning messages regarding noninvertible information matrices, which are probably caused by empirical underidentification (Kenny, 1979). Empirical underidentification occurs when the model should be identified by its structure but it is not identified based on the sample data (Kenny, 1979). With empirical underidentification, fit measures cannot be computed. Notice that only Expanded format scales had this issue; this indicates a model with a method factor is not suitable for the Expanded format scales (i.e., the Expanded format scales are not affected by method effect).

Reference:

Kenny, D. A. (1979). Correlation and Causality. New York: Wiley.
